# Uncovering potential downstream targets of oncogenic GRPR overexpression in prostate carcinomas harboring ETS rearrangements

**DOI:** 10.18632/oncoscience.142

**Published:** 2015-03-17

**Authors:** Joana Santos, Diana Mesquita, João D. Barros-Silva, Carmen Jerónimo, Rui Henrique, António Morais, Paula Paulo, Manuel R. Teixeira

**Affiliations:** ^1^ Department of Genetics and Cancer Genetics Group – CI-IPOP, Portuguese Oncology Institute-Porto, Rua Dr. António Bernardino de Almeida, Porto, Portugal; ^2^ Cancer Biology and Epigenetics Group – CI-IPOP, Portuguese Oncology Institute-Porto, Rua Dr. António Bernardino de Almeida, Porto, Portugal; ^3^ Department of Pathology, Portuguese Oncology Institute-Porto, Rua Dr. António Bernardino de Almeida, Porto, Portugal; ^4^ Department of Urology, Portuguese Oncology Institute-Porto, Rua Dr. António Bernardino de Almeida, Porto, Portugal; ^5^ Department of Pathology and Molecular Immunology, Institute of Biomedical Sciences Abel Salazar (ICBAS), University of Porto, Rua de Jorge Viterbo Ferreira, Porto, Portugal

**Keywords:** prostate cancer, ETS positive tumors, GRPR overexpression, target genes, oncogenic role

## Abstract

Gastrin-releasing peptide receptor (GRPR) is known to be overexpressed in several human malignancies, including prostate cancer, and has been implicated in multiple important neoplastic signaling pathways. We recently have shown that GRPR is an *ERG* and *ETV1* target gene in prostate cancer, using a genome-wide scale and exon-level expression microarray platform. Due to its cellular localization, the relevance of its function and the availability of blocking agents, GRPR seems to be a promising candidate as therapeutic target. Our present work shows that effective knockdown of GRPR in LNCaP and VCaP cells attenuates their malignant phenotype by decreasing proliferation, invasion and anchorage-independent growth, while increasing apoptosis. Using an antibody microarray we were able to validate known and identify new targets of GRPR pathway, namely AKT1, PKCε, TYK2 and MST1. Finally, we show that overexpression of these GRPR targets is restricted to prostate carcinomas harboring *ERG* and/or *ETV1* rearrangements, establishing their potential as therapeutic targets for these particular molecular subsets of the disease.

## INTRODUCTION

Prostate carcinoma (PCa) is the most incident neoplasia in men and the second leading cancer-related cause of death [1]. PCa is a heterogeneous disease and current therapeutic strategies are dependent on TNM staging, Gleason scoring, PSA levels and overall health status. Primary treatment consists mainly of radical prostatectomy and/or radiation therapy, which may be supplemented with androgen ablation [2]. Although many patients are identified with locally, surgically curable, disease, there is a subset of patients that progress or show metastatic prostate cancer, where the gold standard therapy is androgen ablation. Moreover, recurrence is frequent, and many patients develop metastatic disease, for which chemotherapy is only moderately effective [3]. Thus, novel therapeutic approaches to metastatic prostate cancer are needed.

A better understanding of the genetics and molecular pathways involved in prostate carcinogenesis should contribute to the current challenge of identifying promising molecular targets involved in PCa progression. Genomic rearrangements involving members of the ETS family of transcription factors are recurrently found in PCa, with *ERG* and *ETV1* being reported in 50% and 10% of the cases, respectively [4, 5]. ETS members have generally been associated with the regulation of cell growth, proliferation, differentiation, and apoptosis, through activation or repression of target genes [6]. Therapeutic targeting of ETS and other transcription factors has been challenging due to their nuclear localization and molecular embedding in DNA–protein and protein–protein complexes [7, 8]. Therefore, it is important to characterize the downstream molecular targets of these aberrant transcription factors, as some of them may be more amenable to targeted therapy. Using a genome-wide scale and exon-level expression microarray platform, we have shown that *ERG* and *ETV1* regulate both specific and shared target genes in PCa [9]. The most overexpressed gene of our list of shared *ERG* and *ETV1* targets was *GRPR*, which encodes for a membrane-bound gastrin-releasing peptide receptor. GRPR, a member of the G-protein coupled receptor superfamily, is expressed in gastric, respiratory, endocrine, muscle and nervous systems [10]. Both GRPR and its specific ligand GRP (gastrin-releasing peptide), are known to be overexpressed in several human malignancies, including neuroblastoma, lung, breast, pancreatic, colorectal, gastric, esophageal and prostatic cancer [11]. In the prostate, GRPR expression was also detected at high levels in the tumor precursor lesion high-grade prostatic intraepithelial neoplasia (HGPIN) [12].

The discovery of GRPR overexpression in cancer cells led to the test of specific GRP analogues for imaging or targeted therapy [13]. In fact, several reports have described the effect of selective GRPR antagonists on inhibition of tumor growth in numerous models, including prostate cancer cell lines (PC-3, DU-145, MDA-PCa-2b) [14-16], although the associated mechanisms are not yet completely understood. In this context, further knowledge of GRPR biology is of major importance. The link we observed between GRPR overexpression and *ERG* and *ETV1* rearrangements may help understand how the expression of this protein is regulated and, especially, clarify the potential use of GRPR as a therapeutic target for the entire subset of PCa harboring ETS rearrangements. In this study, we aimed to characterize the oncogenic role of GRPR in prostate cancer in an ETS context and to identify specific players involved in the GRPR pathway with potential to be used as therapeutic targets for this particular subset of prostate cancers.

## RESULTS

### *GRPR* is overexpressed in prostate tumors and cell lines harboring *ERG* and *ETV1* rearrangements

**Figure 1:**
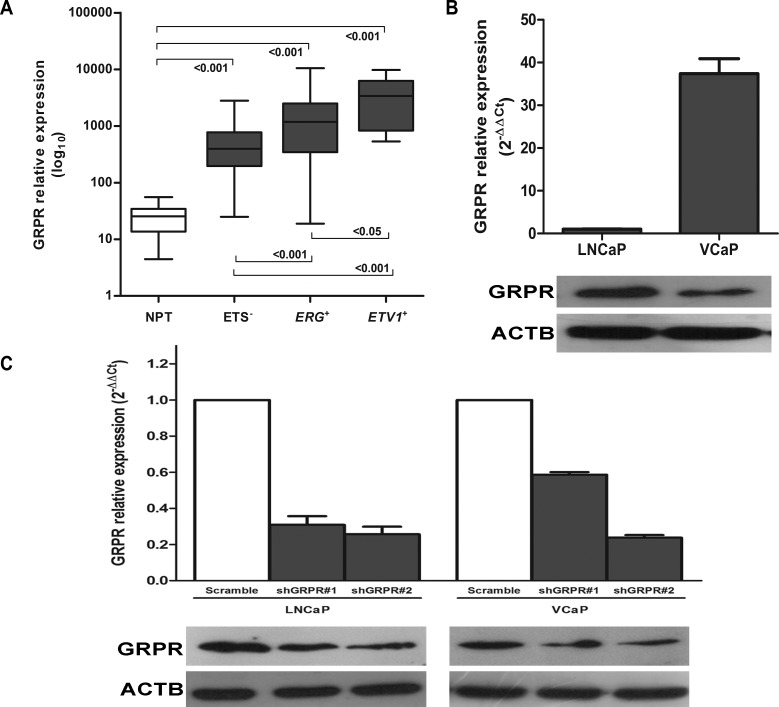
GRPR expression in prostate carcinomas and cell line models of *ERG* and *ETV1* rearrangements (A) Validation of *GRPR* overexpression in a partially-independent series of 160 prostatectomy tumors, including 79 samples with *ERG* rearrangement, 16 samples with *ETV1* rearrangement, and 65 samples without known ETS rearrangements, and 15 morphologically normal prostate tissues (NPT) by Real Time RT-PCR. ETV1+ and ERG+ represent PCa with rearrangements involving *ETV1* and *ERG*, respectively, and ETS-represents PCa negative for known ETS rearrangements. *p*-values of two-group comparisons (MW) are shown. (B) Real Time RT-PCR (top) and immunoblotting (bottom) of *GRPR* expression in the cell line models of *ETV1* and *ERG* rearrangements, LNCaP and VCaP, respectively. (C) Real Time RT-PCR (top) and immunoblotting (bottom) of *GRPR* expression after stable silencing in LNCaP and VCaP cell lines. For each cell line, a negative control (scramble) and two independently silenced cell populations (shGRPR#1 and shGRPR#2) were established.

To validate previous findings showing GRPR overexpression in tumors harboring ETS rearrangements [9], the mRNA expression of *GRPR* was evaluated in a partially-independent series of 160 PCa and 15 morphologically normal prostate tissues (NPT) by real time RT-PCR. We confirmed a statistically significant *GRPR* overexpression in both *ERG* and *ETV1* rearrangement-positive PCa comparing with NPT samples (*p*<0.001) and ETS-negative PCa (*p*<0.001) (Fig.1A). Expression of *GRPR*, both at mRNA and protein levels, was detected in *ERG* and *ETV1* rearrangement-positive prostate cancer cell lines VCaP and LNCaP, respectively (Fig. 1B). For each cell line, two independent silenced populations (shGRPR#1 and shGRPR#2) and a non-targeting control (Scramble) were established. As observed by both real-time RT-PCR and western blot, successful silencing was achieved in both cell lines, allowing a decrease in *GRPR* expression of 60-70% in LNCaP cells and of about 50% in VCaP cells (Fig. 1C).

### Stable knockdown of *GRPR* expression impairs proliferation and promotes apoptosis

**Figure 2:**
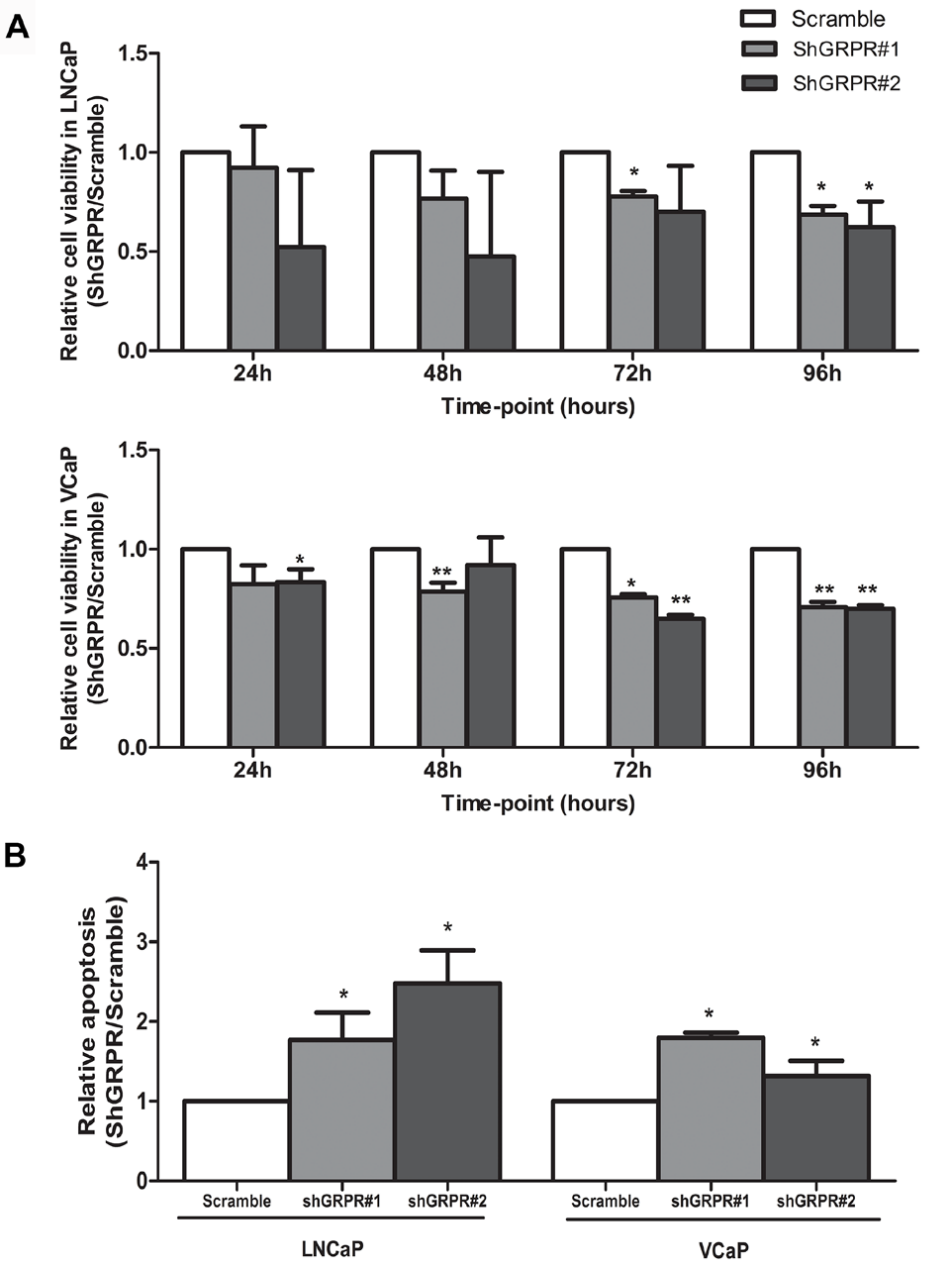
Impact of *GRPR* silencing in LNCaP and VCaP cell lines in cell viability and apoptosis (A) Quantitative analysis of metabolically active cells by the MTT assay, at four time-points. (B) Quantification analysis of apoptotic levels at 96h in culture. For both assays, results are shown for each silenced cell population relative to the scramble cells, from three independent experiments. Statistically significant *p* values are showed by an asterisk (*p<0.05; **p<0.01).

To evaluate the impact of *GRPR* silencing in the acquisition of early-stage characteristics of prostate cancer cells in the context of *ERG* and *ETV1* rearrangements, proliferation and apoptosis were assessed. *GRPR* silenced cell populations (shGRPR) of both cell line models displayed significantly reduced cell viability (Fig. 2A) and increased apoptosis (Fig. 2B), comparing to the corresponding scramble controls. In fact, at 96h in culture, *GRPR* silencing led to a 30% decrease (*p*<0.05) in the number of viable cells in both cell lines, and to a 2 and 1.5-fold increase (*p*<0.05) in apoptosis levels, in LNCaP and VCaP cells, respectively.

### GRPR is involved in the activation of invasion and anchorage independent properties *in vitro*

**Figure 3:**
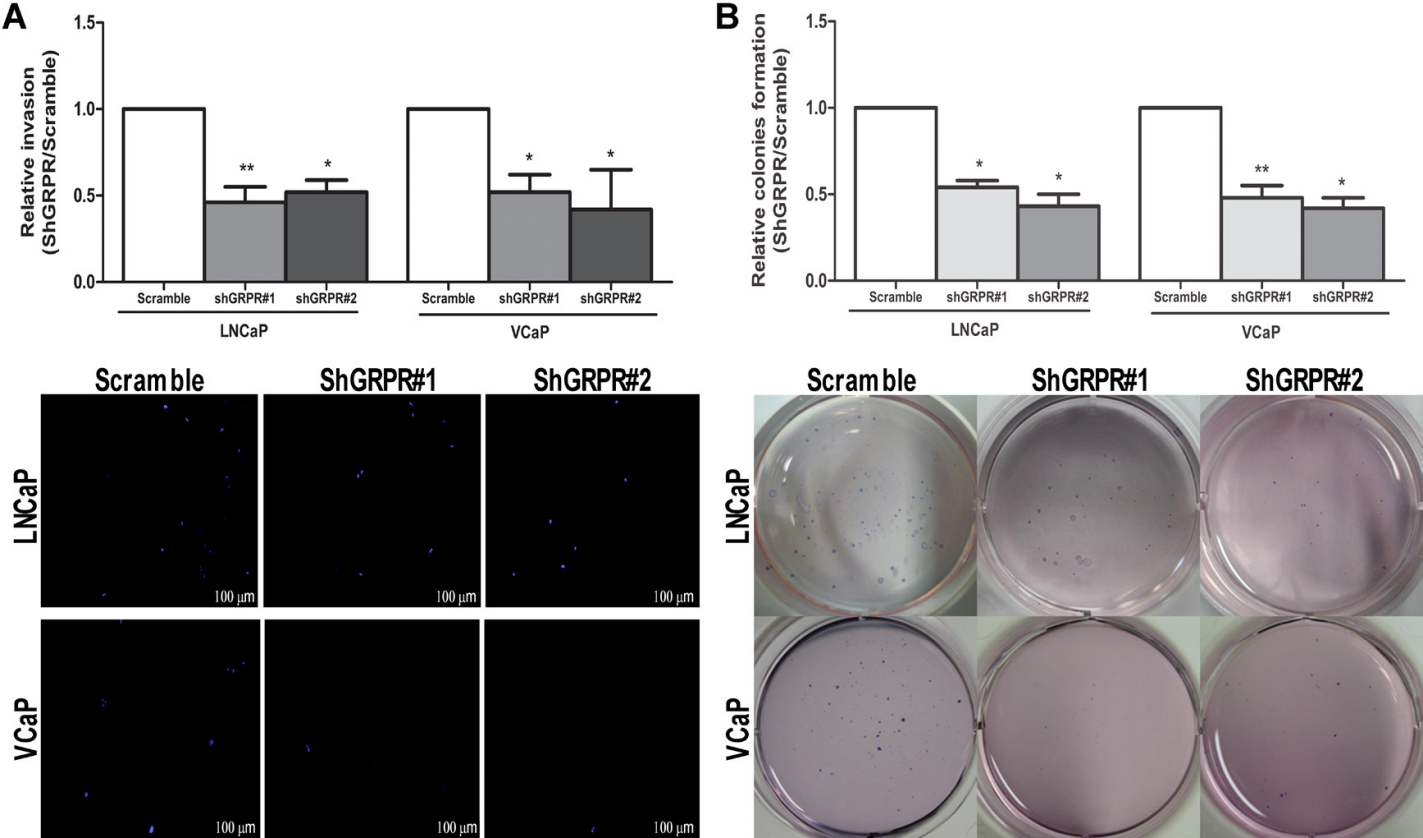
Impact of *GRPR* silencing in LNCaP and VCaP cell lines in invasion and anchorage-independent growth (A) Quantitative analysis (top) and qualitative visualization (bottom) of cell invasion using Matrigel Invasion Chambers. (B) Quantitative analysis (top) and qualitative visualization (bottom) of anchorage-independent growth by the Soft agar colony formation assay. Results are shown for each silenced cell population relative to the scramble cells from three independent experiments. Statistically significant *p* values are showed by an asterisk (*p<0.05; **p<0.01).

To evaluate whether GRPR could be involved in the phenotypic characteristics of advanced prostate cancer cells, we evaluated the impact of *GRPR* silencing in invasion potential and in the capacity to grow without attachment. Using the *in vitro* Matrigel invasion assay, and comparing to scrambled cells, shGRPR cell populations from both cell lines showed a significant reduction of their invasion ability (around 50% decrease, *p*<0.05) (Fig. 3A). Similarly, looking at the capacity of cells to grow without attachment, we found that cell populations with stable *GRPR* silencing developed about 50% fewer colonies than scrambled controls (*p*<0.05) (Fig. 3B).

### Identification of potential *in vitro* GRPR downstream targets by antibody microarray

**Figure 4:**
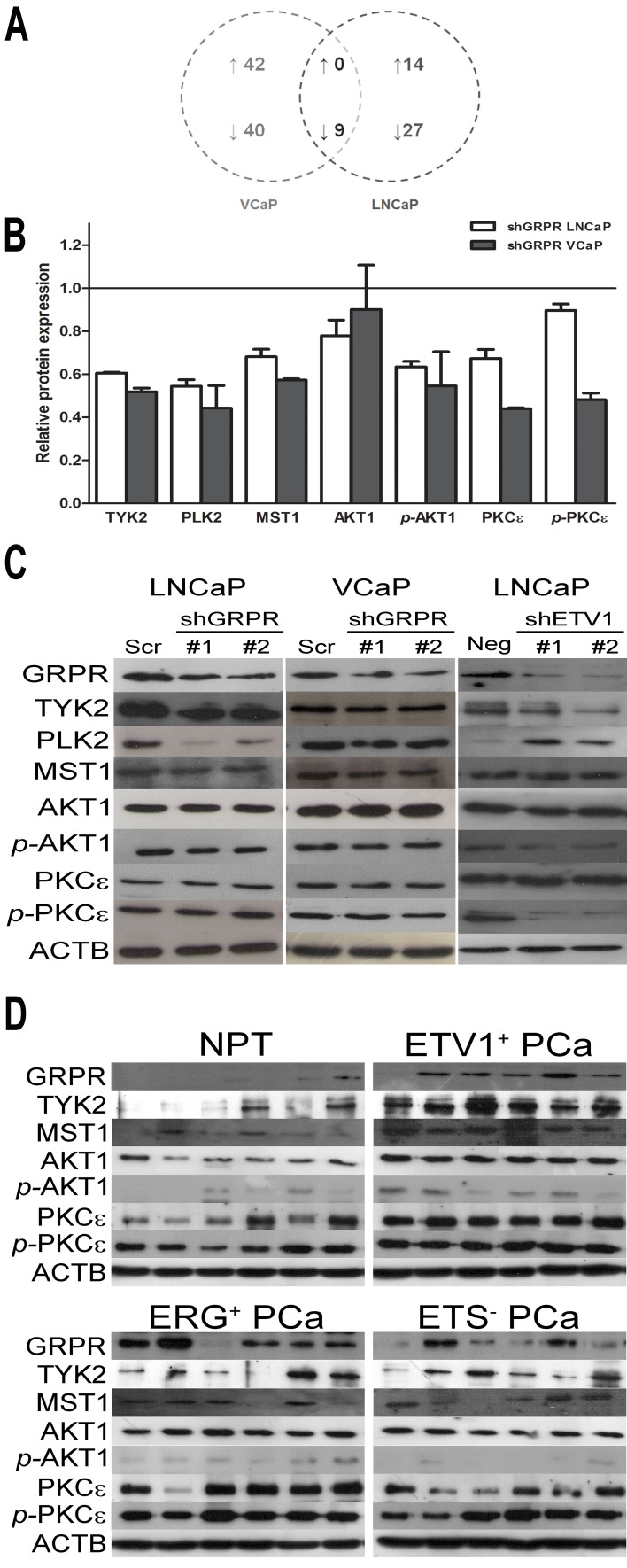
Dissection of potential GRPR downstream targets (A) Venn-diagram of the number of significantly deregulated proteins (at least 1.2-fold) in GRPR silenced cell populations from LNCaP and VCaP cell lines, showing specific and shared deregulated candidate targets (Supplementary Table 2). (B) Relative protein expression of potential targets of GRPR in LNCaP and VCaP cell lines, revealed by KAM-850 antibody microarray. Globally normalized protein expression was compared between shGRPR and scramble (relative protein expression: 1.0) for each cell line, and shared targets of silenced GRPR cell lines were selected after Z score calculation. (C) Immunoblotting validation of previously selected down-regulated targets of GRPR in LNCaP and VCaP cell line models of GRPR silencing and in the LNCaP model of ETV1 silencing. (Scr – scramble; Neg - negative). (D) Immunoblotting analysis of previously selected down-regulated targets of GRPR in protein extracts of ETS-subtyped prostate tumors. ETV1+ and ERG+ represent PCa with rearrangements involving *ETV1* and *ERG*, respectively, ETS-represents PCa negative for known ETS rearrangements and NTP represents morphologically normal prostate tissues.

To discover potential downstream targets of GRPR, we used KAM-850, an antibody microarray that features 850 specific antibodies (Supplementary Table 1). This platform was used to compare the differential protein expression pattern between scrambled and shGRPR populations in both LNCaP and VCaP cell line models. In order to find potential oncogenes regulated by GRPR that could be interesting for targeted therapy of PCa with ETS rearrangements, we focused on down-regulated targets shared by both cell lines (Fig. 4A, Supplementary Table 2). Through this analysis we found a list of nine proteins with decreased expression levels in both cell lines and, based on their cell pathways association, we focused our attention in five of them (Fig. 4B): PLK2, TYK2, MST1, *p*-AKT1 (Ser473) and *p-*PKCε (Ser729). We also included in the remaining analysis pan-AKT1 and pan-PKCε in order to detected total AKT and PKCε protein. To validate these results, western blot analysis was performed using RIPA protein extracts (Fig. 4C). We confirmed that *GRPR* silencing leads to a decreased expression of TYK2, PLK2, MST1 and *p-*AKT1 in both LNCaP and VCaP cells. Total PKCε and *p-*PKCε showed a small decrease in expression only in shGRPR-VCaP cells, and no changes were observed in the expression of total AKT1 in both cell line models.

### *In vivo* validation of *in vitro* GRPR downstream targets

To evaluate whether the *in vitro* association between the expression of GRPR and of these potential targets under an ETS-rearrangement context would be observed *in vivo*, protein extracts of six NPT and 18 PCa (six of each ETS subgroup), randomly selected, were analyzed by western blot (Fig. 4D). This approach showed that, overall, the expression of AKT1 was higher in PCa samples when compared with NPT. Interestingly, tumors with *ETV1* rearrangement showed consistently higher expression of TYK2, MST1 and *p*-AKT1, when compared with both NPT and other PCa subgroups, in which the expression pattern of those proteins showed to be highly heterogeneous. Regarding PKCε and *p-*PKCε expression, both *ETV1* and *ERG* rearrangement-positive PCa samples showed consistently higher expression when compared with NPT, although high protein levels were also detected in some ETS-negative PCa. We were unable to detect PLK2 expression in prostate tissues using two different primary antibodies. Considering the stronger association between the expression of some of the *in vitro* identified GRPR candidate target genes and the presence of *ETV1* rearrangements in PCa samples, we thought to investigate that association using our previously established model of *ETV1* silencing in LNCaP cells. We observed that *ETV1* silencing leads to a decrease in GRPR protein levels (as expected [9]), but also to a decrease in the expression of TYK2, MST1, *p*-AKT1 and *p*-PKCε (Fig. 4C). In agreement with the data observed for cell populations with GRPR silencing, immunoblotting of *ETV1* silenced LNCaP cells did not show differences in AKT1 and PKCε expression. Contrarily, higher PLK2 expression was observed in shETV1-LNCaP cells.

## DISCUSSION

Following our previous work that provided the identification of potential target genes regulated by both *ERG* and *ETV1* transcription factors in PCa, we focused our attention in *GRPR*, the top-most differentially expressed gene of a list of 27 ETS candidate targets [9]. Due to its cellular localization, the relevance of its function and the availability of blocking agents, GRPR seems to be a promising candidate for targeted therapy. Several emergent studies point to the potential of GRPR as a therapeutic target, supporting its role as an important player of signaling pathways in cancer cells, namely cell proliferation, metastasis and angiogenesis [17]. After validating a higher expression of *GRPR* in PCa samples harboring either *ERG* or *ETV1* rearrangements, we decided to evaluate the phenotypic impact of this receptor *in vitro* by knocking down its expression in either *ERG*- or *ETV1*-rearranged prostate cell lines (VCaP and LNCaP, respectively). Upon successful and stable *GRPR* silencing in both cell lines, we observed a decline of malignant cells' phenotype through reduction of cell proliferation, invasion and ability to growth in the absence of cell attachment, and by an increment of apoptosis. Although GRPR and its specific peptide have been associated with an oncogenic role in different tissues and models, the present work is the first report ascertaining the malignant impact of this receptor in prostate carcinogenesis. The observed phenotypic effects and the lack of proved efficacy of GRPR antagonists as therapeutic approaches [18], prompted us to look for potential GRPR target proteins using an antibody microarray, focusing on relevant cellular pathways frequently deregulated in tumorigenesis. Multiple molecular pathways are involved in the proliferation and survival of prostate cancer cells during tumor progression. Among these survival-signaling pathways, up-regulation of the PI3K/Akt pathway is particularly important, considering its role in survival enhancement and apoptosis inhibition [19]. Other authors reported that GRP can induce Akt phosphorylation at Serine 473 in a non-small cell lung carcinoma cell line, and that this activation occurred through transactivation of the epidermal growth factor receptor (EGFR), a known Akt activator [20]. In this work, we observed a significant increase in apoptosis levels and a reduction of cell viability after GRPR knockdown, eventually as a result of disturbing PI3K/Akt pathway via down-regulation of *p*-AKT1 (Ser473). Considering the increased levels of *p*-AKT1 (Ser473) observed in tumors harboring ETS rearrangements, these observations support the hypothesis that ETS overexpression up-regulates the expression of GRPR and subsequently leads to up-regulation of *p*-AKT1 (Ser473), placing ETS transcription factors as upstream regulators of GRPR overexpression in PCa. Interestingly, PKCε, a protein kinase described to be overexpressed in most solid tumors (including those of the prostate) and to have crucial roles in several aspects of tumor development, namely cell transformation, proliferation, cancer cell survival, EMT, migration and invasion [21, 22], was also found overexpressed in our ETS-positive tumors. In our cell line models, PKCε seemed to be more dependent of the ETS context than of the GRPR overexpression, as no significant effect was observed on PKCε/*p*-PKCε expression upon silencing of *GRPR* in both cell line models (VCaP and LNCaP) but a significant decrease of *p*-PKCε was observed in LNCaP cells upon silencing of *ETV1*. In fact, *p*-PKCε was identified as the active kinase that phosphorylates AKT1 at serine 473 leading to full AKT activation [23]. We therefore suggest a link between ETS overexpression and increased PKCε/*p*-PKCε expression, as a GRPR alternative mediator of *p*-AKT1 (Ser473) activation. These findings are in agreement with studies proposing that high levels of ETS protein collaborate with constitutively activated AKT kinase, leading to the development of more aggressive PCa [24].

Additionally, our work revealed that GRPR plays an important role in anchorage-independent growth and invasion in the human prostate cancer cell lines tested, as GRPR silencing led to a significantly decrease in the invasive capacity of both LNCaP and VCaP cell lines. This effect could be the result of down-regulation of TYK2 and MST1 expression, as observed by immunoblotting of GRPR silenced populations from both cell lines. In fact, overexpression of TYK2 (a member of the Janus family of non-receptor tyrosine kinases, JAKs) has been described in several malignancies, such as PCa and squamous cervical carcinomas, as well as in breast cancer cell lines [25], with some studies showing its involvement in enhancing prostate cancer invasion [26, 27]. Similarly, the signaling initiated by the binding of MST1 to its receptor (MST1R) is an important pathway for invasive growth in different neoplasias [28]. However, we were only able to detect strong expression of both TYK2 and MST1 proteins in *ETV1*-positive PCa, and silencing of *ETV1* in LNCaP cells (which also leads to a drastic impair of both cell invasion and anchorage-independent growth [29]) only showed a significant effect in the expression of TYK2, but not in MST1. These observations may indicate that both GRPR and ETV1 may regulate the expression of TYK2 and MST1, which potentially act cumulatively when overexpression of both is present. Taken together, these data support the hypothesis that targeting TYK2 and/or MST1 with specific inhibitors could be a useful approach in the blockage of prostate cancer progression in *ETV1*- positive PCa.

A decrease in PLK2 expression was also observed in our *GRPR* silenced cell populations (with higher impact in LNCaP cells), however the opposite effect was observed in response to *ETV1* silencing in the LNCaP cell line. This suggests that PLK2 expression levels would be the result of a balance between the two factors, with ETV1/ETS transcription factors acting as repressors and GRPR as an activator. Nevertheless, no information was obtained from our series of prostate tissues, since PLK2 expression was not detected in any of the samples analyzed using two different antibodies. This observation, however, is in accordance to the low PLK2 expression levels described for normal and tumorous prostate tissues (Supplementary Figure 1), suggesting that PLK2 expression levels in cell lines may result from adaptation to *in vitro* conditions, and further reflect the relevance of looking into tumor samples to validate *in vitro* associations.

**Figure 5:**
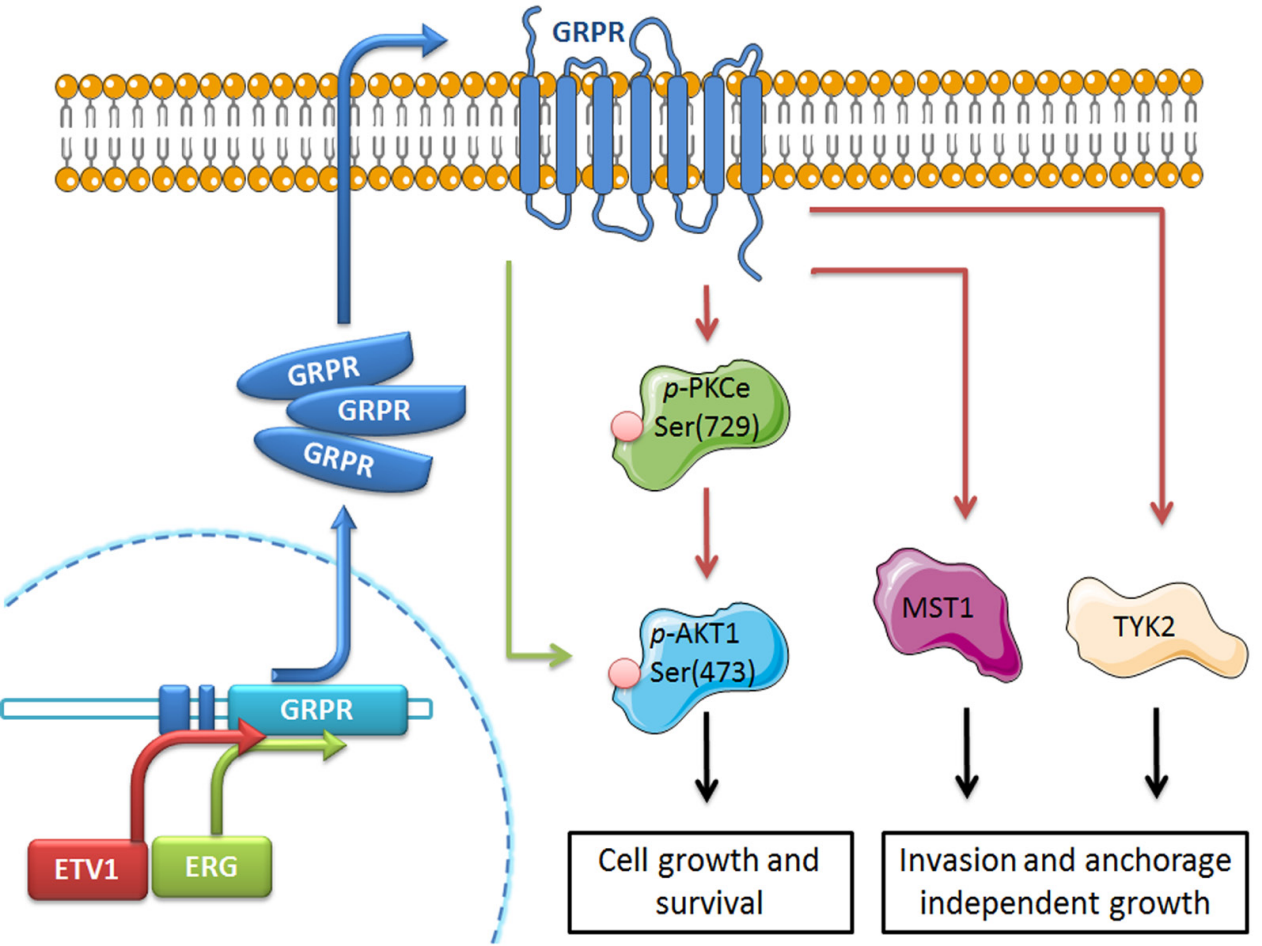
Proposed model for GRPR involvement in the acquisition of oncogenic properties of prostate cancer cells harboring ETS rearrangements Overexpression of ETV1 or ERG in prostate cancer cells (as those harboring *ETV1* or *ERG* rearrangements) increases the transcription of the GRPR gene and consequently leads to overexpression of the GRPR protein. As a G-protein coupled receptor, overexpressed GRPR leads to an increased expression/activation of targets known to be involved in particular cancer pathways, namely AKT1, PKCε, MST1 and TYK2. These deregulated proteins thus constitute promising therapeutic targets for these particular cancer subsets. Green and red arrows represent activation of targets associated with ERG and ETV1, respectively.

In this study, we report the oncogenic role of GRPR in different biological processes of prostate cancer progression through activation of specific targets involved in cancer-associated signaling pathways (including PI3K/Akt and JAK-STAT). Besides validating GRPR as a potential target gene of both *ERG* and *ETV1* transcription factors, our data reveal the activation of different intermediate players of GRPR/ETS *in vitro*-mediated proliferation/apoptosis and invasion/anchorage-independent growth, associated with disease aggressiveness. Considering what is known concerning the activity of these intermediates and the data shown here, we propose a model for GRPR signaling under an ETS-rearrangement cellular context (Fig. 5), where TYK2, MST1 and *p*-Akt may constitute promising therapeutic targets that should be explored in combination with GRPR inhibitors for treating these particular subtypes of prostate cancer.

## MATERIAL AND METHODS

### Tissue samples

We used a series of prostate carcinomas previously characterized for ETS rearrangements [30] and selected 160 samples to represent the various molecular subtypes of PCa, including 79 samples with *ERG* rearrangement, 16 samples with *ETV1* rearrangement, and 65 samples without known ETS rearrangements. As control samples, 15 morphologically normal prostate tissues (NPT) were used (collected from peripheral zones of non-cancerous prostate from bladder cancer patients submitted to cystoprostatectomy) [9]. The groups of *ETV1*-positive PCa and NPT samples included the 13 and nine samples, respectively, which we had previously analyzed by expression microarrays [9]. This study was approved by the institutional review board.

### Cell lines and reagents

The human prostate cell lines used in this study were LNCaP and VCaP. Both cell lines were maintained in standard growth medium, supplemented with 10% fetal bovine serum (Gibco by Life Technologies, Carlsbad, CA, USA) and 1% penicillin/streptomycin solution (Gibco) in a humidified chamber (37°C, 5% CO ). LNCaP cells were acquired from the German Resource Centre for Biological Material (DSMZ, Braunschweig, Germany) and VCaP cells from the European Collection of Cell Cultures (Sigma-Aldrich, St Louis, MO). For validation purposes, both prostate cell lines were karyotyped by G banding and probed for *ERG* and *ETV1* rearrangements by FISH analysis. Cultures were considered *Mycoplasma-*free by routine testing for *Mycoplasma spp.* contamination (PCR Mycoplasma Detection Set; Clontech Laboratories Inc., Mountain View, CA, USA).

### RNA isolation, cDNA synthesis and real-time RT- PCR

Total RNA extraction from tissue samples with TRIzol (Invitrogen by Life Technologies) was previously described [30]. For cDNA synthesis, 200 ng of RNA and the TransPlex Whole Transcriptome Amplification Kit (Sigma-Aldrich) were used, following the manufacturer's instructions. For cell lines, total RNA was extracted using the Illustra TriplePrep Kit (GE Healthcare Bio-science Corporation, NJ, USA), and cDNA was obtained from 1μg of RNA using oligo-dT primers and the H-minus RevertAid cDNA synthesis kit (Fermentas, Ontario, Canada), according to the manufacturer's instructions. Real-time RT-PCR was performed using pre-developed TaqMan^®^ Gene Expression assays (Applied Biosystems, Foster City, CA, USA). Amplification reactions were carried out in triplicates on a 7500 Sequence Detection System (Applied Biosystems), with *GUSB* used as a reference gene. Relative expression was obtained using the comparative Ct method [31].

### *GRPR* and *ETV1* stable silencing

The expression of GRPR was stably silenced in the prostate cancer cell lines (LNCaP and VCaP) by specific short-hairpin RNAs (GRPR shRNA; sc-106924-V) and the shRNA Lentiviral Particles Transdution System, both from Santa Cruz Biotechnology Inc. (CA, USA). A negative scrambled shRNA lentiviral particle (sc-108080) was used to generate a biological control. Cells were plated in a 12-well plate to reach 50%-70% confluence on the day of infection. The lentiviral particles were used to infect the prostate cancer cell lines after addition of polybrene (4.0 μg/ml, Sigma-Aldrich). Effectively transfected cells grew under selective pressure by Puromycin dihydrochloride (cat. 631306, Clontech Laboratories Inc.) at 2.5 μg/ml. The LNCaP cell line model with stable *ETV1* silencing (LNCaP-shETV1 and LNCaP-shNeg populations) was previously established [9].

### Protein extraction and Western blotting

Protein was extracted from sub-confluent cell lines using RIPA lysis buffer (sc-24948, Santa Cruz Biotechnology Inc.) and from tissue samples using the organic fractions obtained after RNA separation with TRIzol (Invitrogen), according to the manufacturer's instructions. Protein concentration was measured using the bicinchoninic acid (BCA) protein assay kit from Thermo Scientific (Waltham, MA, USA) and 40 μg or 10ug (cell lines or tissues, respectively) of total protein were loaded in 10% (w/v) Bis-Tris–containing polyacrylamide gels under reducing conditions for SDS-PAGE. After proteins transfer to a nitrocellulose membrane (Merck Millipore, Billerica, MA, USA), blots were blocked with 5% fat- free milk (Bio-Rad Laboratories, Hercules, CA, USA) in TBS-T (50 mM Tris, 150 mM NaCl, 0.1% Tween-20, pH 7.4) and incubated with primary antibodies at 4°C overnight. Blots were then incubated with a horseradish peroxidase–conjugated secondary antibody for 1h at room temperature, and developed with the enhanced chemiluminescence Western blotting detection system Immun-Star™ WesternC™ Kit (Bio-Rad Laboratories), according to the manufacturer's indications. The primary antibodies used were: rabbit anti-GRPR (ab39883, 1:500, Abcam, Cambridge, UK); rabbit anti-phospho-AKT (Ser473) (bs-0876R, 1:10000, Bioss Inc., MA, USA); rabbit anti-phospho-PKCε (Ser729) (06-821-I, 1:2000, Merck Millipore); and rabbit anti-PKCε (sc-214, 1:2000), goat anti-MST1 (N-19) (sc-6213, 1:100), goat anti-PLK2 (C-18) (sc-9577, 1:100), rabbit anti-TYK2 (sc-169, 1:2000) and mouse anti-AKT1 (B-1) (sc-5298, 1:10000), all purchased from Santa Cruz Biotechnology Inc. A mouse anti-β-actin monoclonal antibody (A1978, 1:8000, Sigma-Aldrich) was used as loading control.

### Cell proliferation assay

The MTT (3-(4, 5-dimethylthiazol-2-yl)-2, 5-diphenyltetrazolium bromide) assay was used for cell viability measurement. LNCaP (1.0 × 10^4^) and VCaP cells (2.5 × 10^4^) were seeded in 96-well plates (Sarstedt AG & Co, Nümbrecht, Germany) in 200 μL of complete growth medium and incubated in a humidified chamber (37°C and 5% CO ). Cells were allowed to adhere and then viability assay was performed at different time-points (24h – 96h). At each time-point, growth medium was replaced by medium containing MTT at 1.0 mg/mL (Sigma-Aldrich) and cells incubated for 1 hour in a humidified chamber. MTT-containing medium was removed and formazan crystals were dissolved using DMSO (Sigma-Aldrich). Finally, plates were shaken for 15 minutes for complete homogenization and absorbance levels were measured at 540 nm with background correction at 630 nm using a microplate reader (Fluostar Omega, BMG Labtech, Offenburg, Germany). For each time-point, an average value of measurements from nine replicate wells was obtained. Cell viability was estimated by correcting and normalizing the average absorbance values obtained in each time-point (Tn) to the average absorbance values of the time zero (T0) by the following formula: (Tn-T0)/T0. Relative cell viability was obtained by normalizing values of each silenced cell population to its respective control. Three independent assays were performed.

### Apoptosis assay

Apoptosis assay was performed according to the manufacturer's instructions (Biocolor, Newtownabbey, Northern Ireland). The APOPercentage assay is a dye- uptake assay which stains only apoptotic cells with a red dye, whereas normal and necrotic cells remain unlabeled. Cells were seeded in 96-well plates at a density of 1.0 × 10^4^ cells (LNCaP) or 2.5 × 10^4^ cells (VCaP) for 96h. Cells were then stained with APOPercentage dye for 1 hour and washed twice with PBS to remove non-cell bound dye. Dye Release Reagent was added to each well and the plate was shaken for 10 minutes. Absorbance levels were measured at 550 nm with background correction at 620 nm using a microplate reader (Fluostar Omega). An average value of measures from nine replicate wells was obtained for each cell population. Relative apoptosis was obtained by normalizing values of each silenced cell population to its respective control. Three independent assays were performed.

### Invasion assay

Cell invasion through a three-dimensional extracellular matrix was evaluated by a Matrigel invasion assay using BD Matrigel Invasion Chambers with 8.0 μm pore (BD Biocoat, Bedford, MA, USA). Briefly, the matrigel-coated transwell chambers were rehydrated and 2.5 × 10^4^ LNCaP cells or 5.0 × 10^4^ VCaP cells in 500 μL of serum-free medium were plated in the upper chamber, in triplicate wells. Complete growth medium was added to the lower chamber. After 48 or 72 hours (LNCaP and VCaP, respectively), the cells on the upper surface were removed with cotton swabs, and invaded cells at the lower surface were fixed with methanol, stained with DAPI and counted under a microscope. Relative cell invasion was obtained by normalizing values of each silenced cell population to its respective control. Three independent assays were performed.

### Soft agar colony formation assay

LNCaP and VCaP cells (1.0 × 10^4^ or 5.0 × 10^4^, respectively) were resuspended in 0.2% low melting agarose in complete growth medium and plated on top of 1 ml underlayer of 0.6% low melting agarose in the same medium in 6-well cell culture plates. After 4 weeks of incubation in a humidified chamber, colonies were stained with 0.05% crystal violet, photographed and counted. Relative aggregation was obtained by normalizing values of each silenced cell population to its respective control. Three independent assays were performed.

### Kinex^™^ antibody microarray (KAM-850)

To perform the Kinex^™^ analyses (Kinexus Bioinformatics Corporation, Vancouver, Canada), 50 μg of total protein lysate from each sample were covalently labeled with a proprietary fluorescent dye and incubated on the chip array. The six protein extracts analyzed, from the *GRPR* silenced cell line models of LNCaP and VCaP cells (GRPR sh#1, GRPR sh#2 and scramble control), were obtained using the kinexus protein lysis buffer (Kinexus Bioinformatics Corporation). The KAM-850 antibody microarray contains over 850 antibodies among pan- and phospho site-specific with wide coverage of cell signaling proteins and pathways. Each array produces a pair of 16-bit images, which were captured with a ScanArray Reader laser array scanner (Perkin-Elmer, Waltham, MA, USA). Image capture, signal quantification, background correction and Z score transformation [32] were performed by Kinexus Bioinformatics Corporation. A Z ratio of ±1.2 was inferred as significant.

### Statistical analysis

All *in vitro* data were obtained from three independent experiments, each including triplicate wells per condition. Statistical analyses were conducted using SPSS software version 21.0 (IBM-SPSS Inc., Chicago, IL, USA) and graphs were built using GraphPad Prism 5.0 software (GraphPad Software Inc., La Jolla, CA, USA). *In vitro* studies data were analyzed by paired Student's *t* test. Kruskal-Wallis test (KW) and Mann-Whitney U test (MW) were used for multi-group comparisons, as appropriate. All *p* values were based on two-sided hypothesis testing and a *p*<0.05 was considered statistically significant.

## SUPPLEMENTARY MATERIALS FIGURES AND TABLES


