# Supplementary material for: Uncovering potential downstream targets of oncogenic GRPR overexpression in prostate carcinomas harboring ETS rearrangements
Source: Oncoscience. 2015 Mar 17;2(5):497–507. doi: 10.18632/oncoscience.142 (PMC4468336; doi:10.18632/oncoscience.142)
Supplement: Supplementary file 1 [file oncoscience-02-0497-s001.pdf]

Uncovering potential downstream targets of oncogenic GRPR overexpression in prostate carcinomas harboring ETS rearrangements

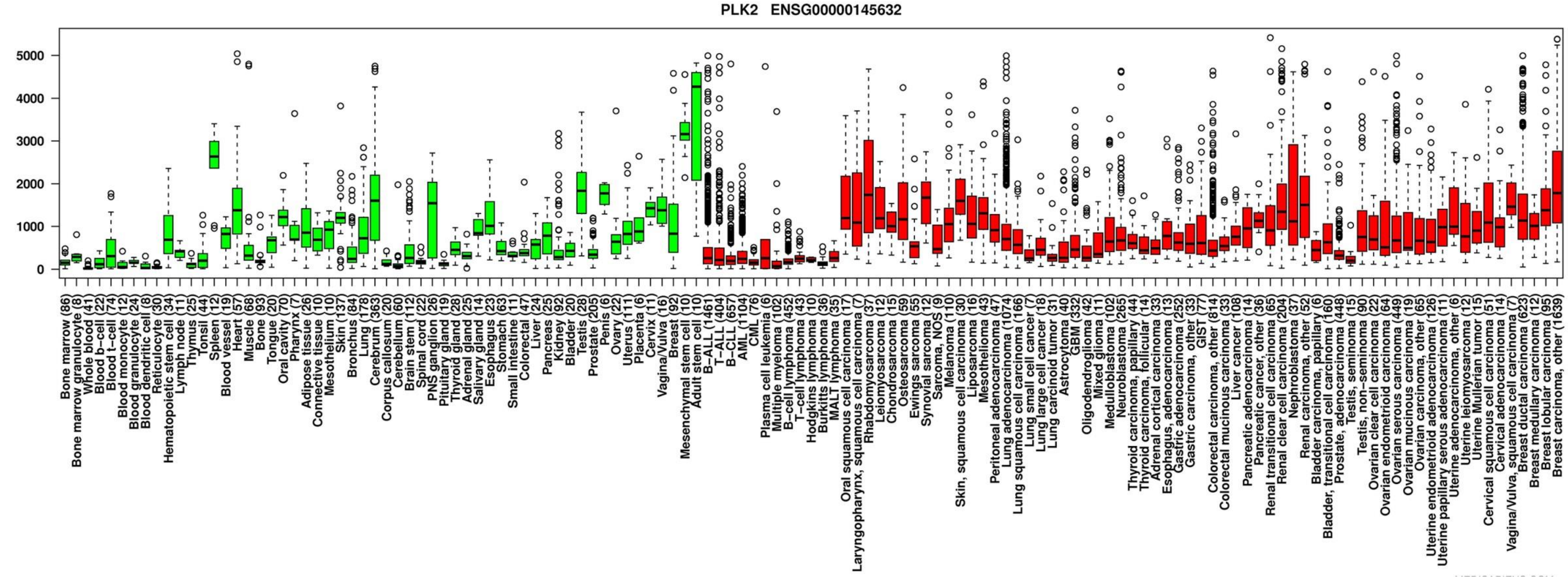

Supplementary Figure 1: Box-whisker plot of the PLK2 gene's expression (ENSG00000145632) in healthy and cancer tissues in IST (in silico transcriptomics) online. Green boxes indicate healthy tissues, red boxes indicate cancers.

Supplementary Table 1: List of the 850 antibodies among pan- and phospho site-specific included in the antibody microarray KAM-850 Kinexus.

No.	Antibody Codes	Target Protein Name	Phospho Site (Human)	Full Target Protein Name	Refseq	Uniprot Link
1	NN001	14-3-3 z	Pan-specific	14-3-3 protein zeta (cross-reacts with other isoforms)	NP_003397	P63104
2	NN166	4E-BP1	Pan-specific	Eukaryotic translation initiation factor 4E binding protein 1 (PHAS1)	NP_004086	Q13541
3	PN001	4E-BP1	S65	Eukaryotic translation initiation factor 4E binding protein 1 (PHAS1)	NP_004086	Q13541
4	PN114	4E-BP1	T45	Eukaryotic translation initiation factor 4E binding protein 1 (PHAS1)	NP_004086	Q13541
5	PN128	4E-BP1	T70	Eukaryotic translation initiation factor 4E binding protein 1 (PHAS1)	NP_004086	Q13541
6	NK001	Abl	Pan-specific	Abelson proto-oncogene-encoded protein-tyrosine kinase	NP_005148	P00519
7	PN002	AcCoA carboxylase	S80	Acetyl coenzyme A carboxylase	NP_000655	Q13085
8	NN135-1	Acetylated Lysine	Pan-specific	Acetylated Lysine	NA	NA
9	NN135-2	Acetylated Lysine	Pan-specific	Acetylated Lysine	NA	NA
10	NK002	ACK1	Pan-specific	Activated p21cdc42Hs protein-serine kinase	NP_005772	Q07912
11	PN003-PN004	Adducin a	S726	Adducin alpha (ADD1)	NP_058432	P35611
12	NN002	AIF	Pan-specific	Apoptosis inducing factor (programed cell death protein 8 (PDCD8))	NP_004199	O95831
13	NN003	AK2	Pan-specific	Adenylate kinase 2	NP_001616	P54819
14	NK003	ALK	Pan-specific	Anaplastic lymphoma kinase	NP_004295.2	Q9UM73
15	NK004	ALS2CR7 (PFTAIRE2)	Pan-specific	Amyotrophic lateral sclerosis 2 chromosomal region candidate gene protein-serine kinase 7	NP_631897	Q96Q40
16	PK002	AMPKa1/2	T183	5'-AMP-activated protein kinase subunit alpha 1/2	NP_006242	Q13131
17	NK006	ANKRD3	Pan-specific	Ankyrin repeat domain protein-serine kinase 3 (RIPK4, DIK)	NP_065690	P57078
18	NN004	APG1	Pan-specific	Hsp 70-related heat shock protein 1 (osmotic stress protein 94 (OSP94))	NP_055093	O95757
19	NN122	APG2	Pan-specific	Hsp 70-related heat shock protein 4 (HSP70RY)	NP_002145.3	P34932
20	PN189	APP	T668	Amyloid beta A4 protein	NP_000475.1	P05067
21	NN121	Arrestin b1	Pan-specific	Arrestin beta 1	NP_004032	P49407
22	PN133	Arrestin b1	S412	Arrestin beta 1	NP_004032	P49407
23	NK007	ASK1 (MAP3K5)	Pan-specific	Apoptosis signal regulating protein-serine kinase	NP_005914	Q99683
24	NK007-2	ASK1 (MAP3K5)	Pan-specific	Apoptosis signal regulating protein-serine kinase	NP_005914	Q99683
25	PK143	ASK1 (MAP3K5)	S966	Apoptosis signal regulating protein-serine kinase	NP_005914	Q99683
26	NN160	ATF2	Pan-specific	Activating transcription factor 2 (CRE-BP1)	NP_001871	P15336
27	PN006-1	ATF2	T69 + T71	Activating transcription factor 2 (CRE-BP1)	NP_001871	P15336
28	PN115	ATF2	S112	Activating transcription factor 2 (CRE-BP1)	NP_001871	P15336
29	NK008-2	Aurora A (AIK)	Pan-specific	Aurora Kinase A (serine/threonine protein kinase 6)	NP_940835	O14965
30	NK193	Aurora B (AIM-1)	Pan-specific	Aurora Kinase B (serine/threonine protein kinase 12)	NP_004208	Q96GD4
31	NK009	Aurora C (AIK3)	Pan-specific	Aurora Kinase C (serine/threonine-protein kinase 13)	NP_003151	Q9UQB9
32	NK010	Axl	Pan-specific	Axl proto-oncogene-encoded protein-tyrosine kinase	NP_001690	P30530
33	PN008	B23 (NPM)	T199	B23 (nucleophosmin, numatrin, nucleolar protein NO38)	NP_002511	P06748
34	PN008-2	B23 (NPM)	T199	B23 (nucleophosmin, numatrin, nucleolar protein NO38)	NP_002511	P06748
35	PN009	B23 (NPM)	T234/T237	B23 (nucleophosmin, numatrin, nucleolar protein NO38)	NP_002511	P06748
36	PN012-1	Bad	S99	Bcl2-antagonist of cell death protein	NP_004313	Q92934
37	NN000	Bak	Pan-specific	Bcl2 homologous antagonist/killer (BCK2L7)	NP_001179	Q16611
38	NN005	Bax	Pan-specific	Apoptosis regulator Bcl2-associated X protein	NP_620116	Q07812
39	NN006	Bcl2	Pan-specific	B-cell lymphoma protein 2 alpha	NP_000624	P10415
40	NN006-1	Bcl2	Pan-specific	B-cell lymphoma protein 2 alpha	NP_000624	P10415
41	NN007	Bcl-xL	Pan-specific	Bcl2-like protein 1	NP_612815	Q07817
42	NN008	Bcl-xS/L	Pan-specific	Bcl2-like protein 1	NP_612815	Q07817
43	PK164	Bcr	Y177	Breakpoint cluster region protein	NP_004318.3	P11274
44	NN009	Bid	Pan-specific	BH3 interacting domain death agonist	NP_001187	P55957
45	NK011	BLK	Pan-specific	B lymphoid tyrosine kinase	NP_001706	P51451
46	PN013	BLNK	Y84	B-cell linker protein	NP_037446	O75498
47	NK012	BMX (Etk)	Pan-specific	Bone marrow X protein-tyrosine kinase	NP_001712	P51813
48	PK003	BMX (Etk)	Y40	Bone marrow X protein-tyrosine kinase	NP_001712	P51813
49	PN014	BRCA1	S1497	Breast cancer type 1 susceptibility protein	NP_009225	P38398
50	PN116	BRCA1	S1423	Breast cancer type 1 susceptibility protein	NP_009225	P38398
51	NK013	BRD2	Pan-specific	Bromodomain-containing protein-serine kinase 2	NP_005095	P25440
52	NK014	Btk	Pan-specific	Bruton's agammaglobulinemia tyrosine kinase	NP_000052	Q06187
53	PK004	Btk	Y223	Bruton's agammaglobulinemia tyrosine kinase	NP_000052	Q06187
54	NK015	BUB1A	Pan-specific	BUB1 mitotic checkpoint protein-serine kinase	NP_004327	O43683
55	NN174	CA9	Pan-specific	Carbonic anhydrase 9	NP_001207.2	Q16790
56	PN015	Caldesmon	S789	Caldesmon	NP_004333	Q05682
57	NN136-2	Calnexin	Pan-specific	Calnexin	NP_001019820.1.	P27824
58	NN137-1	Calreticulin	Pan-specific	Calreticulin	NP_004334.1.	P27797
59	NK211	CAMK1a	Pan-specific	Calcium/calmodulin-dependent protein-serine kinase 1 alpha	NP_003647.1	Q14012
60	NK016-1	CaMK1d	Pan-specific	Calcium/calmodulin-dependent protein-serine kinase 1 delta	NP_003647	Q8IU85

61	NK016-2	CaMK1d	Pan-specific	Calcium/calmodulin-dependent protein-serine kinase 1 delta	NP_003647	Q8IU85
62	PK005-1	CaMK2a	T286	Calcium/calmodulin-dependent protein-serine kinase 2 alpha	NP_741960	Q9UQM7
63	NK018-2	CAMK2b	Pan-specific	Calcium/calmodulin-dependent protein-serine kinase 2 beta	NP_742081	Q13554
64	NK019-2	CAMK2d	Pan-specific	Calcium/calmodulin-dependent protein-serine kinase 2 delta	NP_742126	Q13557
65	NK021	CaMK4	Pan-specific	Calcium/calmodulin-dependent protein-serine kinase 4	NP_001735	Q16566
66	NK021-2	CaMK4	Pan-specific	Calcium/calmodulin-dependent protein-serine kinase 4	NP_001735	Q16566
67	NK021-3	CaMK4	Pan-specific	Calcium/calmodulin-dependent protein-serine kinase 4	NP_001735	Q16566
68	NK022	CaMKK (CaMKK1)	Pan-specific	Calcium/calmodulin-dependent protein-serine kinase kinase	NP_006540	Q8N5S9
69	NN010	CAS	Pan-specific	Cellular apoptosis susceptibility protein (CSE1L)	NP_001307	P55060
70	NK023	CASK/Lin2	Pan-specific	Calcium/calmodulin-dependent protein-serine kinase (Lin2 homolog)	NP_001119526.1	O14936
71	NN011	CASP1	Pan-specific	Caspase 1 (Interleukin-1 beta convertase)	NP_001214	P29466
72	NN011-2	CASP1	Pan-specific	Caspase 1 (Interleukin-1 beta convertase)	NP_001214	P29466
73	NN013	CASP3	Pan-specific	Caspase 3 (apopain, cysteine protease CPP32)	NP_004337	P42574
74	NN013-3	CASP3	Pan-specific	Caspase 3 (apopain, cysteine protease CPP32)	NP_004337	P42574
75	NN016	CASP6	Pan-specific	Caspase 6 (apoptotic protease Mch2)	NP_001217	P55212
76	NN017	CASP7	Pan-specific	Caspase 7 (ICE-like apoptotic protease 3 (ICE-LAP3), Mch3)	NP_01218	P55210
77	NN017-2	CASP7	Pan-specific	Caspase 7 (ICE-like apoptotic protease 3 (ICE-LAP3), Mch3)	NP_01218	P55210
78	NN019-2	CASP9	Pan-specific	Caspase 9 (ICE-like apoptotic protease 6 (ICE-LAP6), Mch6, APAF3)	NP_033938	P55211
79	PN162	Catenin a	S641	Catenin (cadherin-associated protein) alpha	NP_001894.2	P35221
80	PN166	Catenin b	S33	Catenin (cadherin-associated protein) beta 1	NP_001895	P35222
81	PN167	Catenin b	Y333	Catenin (cadherin-associated protein) beta 1	NP_001895	P35222
82	NN021	Catenin b1	Pan-specific	Catenin (cadherin-associated protein) beta 1	NP_001895	P35222
83	NN021-1	Catenin b1	Pan-specific	Catenin (cadherin-associated protein) beta 1	NP_001895	P35222
84	NN167	Caveolin 1	Pan-specific	Caveolin 1	NP_001744.2	Q03135
85	PN147	Caveolin 1	Y14	Caveolin 1	NP_001744.2	Q03135
86	NN022-1	Caveolin 2	Pan-specific	Caveolin 2	NP_001224	P51636
87	PN018	Caveolin 2	S36	Caveolin 2	NP_001224	P51636
88	PN171	Cbl	Y700	Signal transduction protein CBL	NP_005179.2	P22681
89	NP001	CD45	Pan-specific	Leukocyte common antigen CD45 receptor-tyrosine phosphatase (LCA, T200)	NP_002829	P08575
90	NP002	Cdc25B	Pan-specific	Cell division cycle 25B phosphatase	NP_004349	P30305
91	NP003	Cdc25C	Pan-specific	Cell division cycle 25C phosphatase	NP_001781	P30307
92	PP005	Cdc25C	S216	Cell division cycle 25C phosphatase	NP_002882.3	P30307
93	NK024	CDC2L5 (CHED)	Pan-specific	Cell division cycle 2-like protein-serine kinase 5	NP_003709	Q14004
94	NN023	Cdc34	Pan-specific	Cell division cycle 34 (ubiquitin-conjugating ligase)	NP_004350	P49427
95	NN024	Cdc42	Pan-specific	Cell division control protein 42 homolog	NP_001782	P60953
96	NK025-1	CDK1 (CDC2)	Pan-specific	Cyclin-dependent protein-serine kinase 1	NP_001777	P06493
97	NK025-2	CDK1 (CDC2)	Pan-specific	Cyclin-dependent protein-serine kinase 1	NP_001777	P06493
98	NK025-3	CDK1 (CDC2)	Pan-specific	Cyclin-dependent protein-serine kinase 1	NP_001777	P06493
99	NK025-4	CDK1 (CDC2)	Pan-specific	Cyclin-dependent protein-serine kinase 1	NP_001777	P06493
100	NK025-5	CDK1 (CDC2)	Pan-specific	Cyclin-dependent protein-serine kinase 1	NP_001777	P06493
101	NK025-6	CDK1 (CDC2)	Pan-specific	Cyclin-dependent protein-serine kinase 1	NP_001777	P06493
102	PK006	CDK1/2	T14+Y15	Cyclin-dependent protein-serine kinase 1/2	NP_001777	P06493
103	PK007-1	CDK1/2	Y15	Cyclin-dependent protein-serine kinase 1/2	NP_001777	P06493
104	PK007-2	CDK1/2	Y15	Cyclin-dependent protein-serine kinase 1/2	NP_001777	P06493
105	PK007-3	CDK1/2	Y15	Cyclin-dependent protein-serine kinase 1/2	NP_001777	P06493
106	PK008	CDK1/2	T161	Cyclin-dependent protein-serine kinase 1/2	NP_001777	P06493
107	NK033	CDK10	Pan-specific	Cyclin-dependent protein-serine kinase 10 [PISSLRE]	NP_003665	Q15131
108	NK026-2	CDK2	Pan-specific	Cyclin-dependent protein-serine kinase 2	NP_001789	P24941
109	NK026-3	CDK2	Pan-specific	Cyclin-dependent protein-serine kinase 2	NP_001789	P24941
110	NK026-4	CDK2	Pan-specific	Cyclin-dependent protein-serine kinase 2	NP_001789	P24941
111	NK026-5	CDK2	Pan-specific	Cyclin-dependent protein-serine kinase 2	NP_001789	P24941
112	NK026-6	CDK2	Pan-specific	Cyclin-dependent protein-serine kinase 2	NP_001789	P24941
113	NK026-7	CDK2	Pan-specific	Cyclin-dependent protein-serine kinase 2	NP_001789	P24941
114	NK027	CDK4	Pan-specific	Cyclin-dependent protein-serine kinase 4	NP_000066	P11802
115	NK027-2	CDK4	Pan-specific	Cyclin-dependent protein-serine kinase 4	NP_000066	P11802
116	NK028-1	CDK5	Pan-specific	Cyclin-dependent protein-serine kinase 5	NP_004926	Q00535
117	NK028-2	CDK5	Pan-specific	Cyclin-dependent protein-serine kinase 5	NP_004926	Q00535
118	NK028-4	CDK5	Pan-specific	Cyclin-dependent protein-serine kinase 5	NP_004926	Q00535
119	NK028-5	CDK5	Pan-specific	Cyclin-dependent protein-serine kinase 5	NP_004926	Q00535
120	NK029	CDK6	Pan-specific	Cyclin-dependent protein-serine kinase 6	NP_001250	Q00534
121	NK029-2	CDK6	Pan-specific	Cyclin-dependent protein-serine kinase 6	NP_001250	Q00534

122	NK029-3	CDK6	Pan-specific	Cyclin-dependent protein-serine kinase 6	NP_001250	Q00534
123	PK165	CDK6	Y13	Cyclin-dependent protein-serine kinase 6	NP_001250	Q00534
124	NK030-2	CDK7	Pan-specific	Cyclin-dependent protein-serine kinase 7	NP_001790	P50613
125	NK030-3	CDK7	Pan-specific	Cyclin-dependent protein-serine kinase 7	NP_001790	P50613
126	NK031-2	CDK8	Pan-specific	Cyclin-dependent protein-serine kinase 8	NP_001252	P49336
127	NK031-4	CDK8	Pan-specific	Cyclin-dependent protein-serine kinase 8	NP_001252	P49336
128	NK031-5	CDK8	Pan-specific	Cyclin-dependent protein-serine kinase 8	NP_001252	P49336
129	NK032	CDK9	Pan-specific	Cyclin-dependent protein-serine kinase 9	NP_001252	P50750
130	NK199	CDKL1	Pan-specific	Cyclin-dependent kinase-like 1	NP_004187.2	Q00532
131	NK034	Chk1	Pan-specific	Checkpoint protein-serine kinase 1	NP_001265	O14757
132	NK034-2	Chk1	Pan-specific	Checkpoint protein-serine kinase 1	NP_001265	O14757
133	PK162	Chk1	S280	Checkpoint protein-serine kinase 1	NP_001265	O14757
134	NK035	Chk2	Pan-specific	Checkpoint protein-serine kinase 2	NP_009125	O96017
135	PK119	Chk2	T68	Checkpoint protein-serine kinase 2	NP_009125	O96017
136	NN025	c-IAP1	Pan-specific	Cellular inhibitor of apoptosis protein 1 (baculoviral IAP repeat-containing protein 3, apoptosis inhibitor 2 (API2))	NP_001156	Q13490
137	NK036	CK1d	Pan-specific	Casein protein-serine kinase 1 delta	NP_001884	P48730
138	NK037-1	CK1e	Pan-specific	Casein protein-serine kinase 1 epsilon	NP_001885	P49674
139	NK198	CK1g	Pan-specific	Casein kinase I gamma 1 isoform	NP_071331	Q9HCP0
140	NK040	CK1g2	Pan-specific	Casein protein-serine kinase 1 gamma 2	NP_001310	P78368
141	NK041	CK2a	Pan-specific	Casein protein-serine kinase 2 alpha/ alpha prime	NP_001887	P68400
142	PK167	CK2a	T360/S362	Casein protein-serine kinase 2 alpha/ alpha prime	NP_001887	P68400
143	PN130	c-Myc	T58/S62	Myc proto-oncogene protein	NP_002458.2	P01106
144	NN026	Cofilin 1	Pan-specific	Cofilin 1	NP_005498	P23528
145	PN019	Cofilin 1	S3	Cofilin 1	NP_005498	P23528
146	PN020	Cofilin 2	S3	Cofilin 2	NP_068733	Q9Y281
147	PN148	Connexin 43	S368	Gap junction alpha-1 protein	NP_000156.1	P17302
148	PN022-2	Cortactin	Y466	Cortactin (amplaxin) (mouse)	NP_031829	Q14247
149	NK042	COT	Pan-specific	Osaka thyroid oncogene protein-serine kinase (Tpl2)	NP_005195	P41279
150	NK042-2	COT	Pan-specific	Osaka thyroid oncogene protein-serine kinase (Tpl2)	NP_005195	P41279
151	NN027	COX2	Pan-specific	Cyclo-oxygenase 2 (prostaglandin G/H synthase 2 precursor)	NP_000954	P35354
152	NK043	CPG16/CaMKinase VI	Pan-specific	Serine/threonine-protein kinase DCAMKL1	NP_004725	O15075
153	PN023	CREB1	S129+S133	cAMP response element binding protein 1	NP_004370	P16220
154	PN024	CREB1	S133	cAMP response element binding protein 1	NP_004370	P16220
155	PN024-2	CREB1	S133	cAMP response element binding protein 1	NP_004370	P16220
156	NN149-1	Crystallin aB	Pan-specific	Crystallin alpha B (heat-shock 20 kDa like-protein)	NP_001876	P02511
157	NN149-2	Crystallin aB	Pan-specific	Crystallin alpha B (heat-shock 20 kDa like-protein)	NP_001876	P02511
158	NN149-3	Crystallin aB	Pan-specific	Crystallin alpha B (heat-shock 20 kDa like-protein)	NP_001876	P02511
159	PN025	Crystallin aB	S19	Crystallin alpha B (heat-shock 20 kDa like-protein)	NP_001876	P02511
160	PN110	Crystallin aB	S45	Crystallin alpha B (heat-shock 20 kDa like-protein)	NP_001876	P02511
161	NK044	Csk	Pan-specific	C-terminus of Src tyrosine kinase	NP_004374	P41240
162	NK044-2	Csk	Pan-specific	C-terminus of Src tyrosine kinase	NP_004374	P41240
163	NN028	Cyclin A	Pan-specific	Cyclin A1	NP_003905	P78396
164	NN029	Cyclin B1	Pan-specific	Cyclin B1	NP_114172	P14635
165	PN190	Cyclin B1	S147	Cyclin B1	NP_114172	P14635
166	NN030-1	Cyclin D1	Pan-specific	Cyclin D1 (PRAD1)	NP_444284	P24385
167	NN030-2	Cyclin D1	Pan-specific	Cyclin D1 (PRAD1)	NP_444284	P24385
168	NN031	Cyclin E	Pan-specific	Cyclin E1	NP_001229	P24864
169	PN191	Cyclin E	T395	Cyclin E1	NP_001229	P24864
170	NN032	Cyclin G1	Pan-specific	Cyclin G1	NP_004051	P51959
171	NN033	CytoC	Pan-specific	Cytochrome C	NP_061820	P99999
172	PN026	Dab1	Y198	Disabled homolog 1	NP_066566	O75553
173	NK210	DAPK1	Pan-specific	Death-associated protein kinase 1	NP_004929	P53355
174	NK046	DAPK2	Pan-specific	Death-associated protein kinase 2	NP_055141	Q9UIK4
175	NN034	DAXX	Pan-specific	Death-associated protein 6 (BING2)	NP_001341	Q9UER7
176	NN163	DDIT3(CHOP)	Pan-specific	DNA damage-inducible transcript 3 protein	NP_001181986.1	P35639
177	NN035-NN126	DFF35	Pan-specific	DNA fragmentation factor alpha (ICAD) 35-kDa subunit	NP_004392	O00273
178	NK219	DGKz	Pan-specific	Diacylglycerol kinase zeta	NP_963290	Q13574
179	NK048	DNAPK	Pan-specific	DNA-activated protein-serine kinase	NP_008835	P78527
180	NK048-2	DNAPK	Pan-specific	DNA-activated protein-serine kinase	NP_008835	P78527
181	PN027	Dok2	Y139	Docking protein 2	NP_034201	O60496
182	PN027-2	Dok2	Y139	Docking protein 2	NP_034201	O60496

183	NK050	DRAK2 (STK17B)	Pan-specific	DAP kinase-related apoptosis-inducing protein-serine kinase 2 (STK17B)	NP_004217	O94768
184	NK051	eEF2K	Pan-specific	Elongation factor-2 protein-serine kinase	NP 037434	O00418
185	NN175	EFNA5	Pan-specific	Ephrin-A5	NP_001953.1	P52803
186	PN173	EFNB2	Y316	EPH-related receptor tyrosine kinase ligand 5	NP_004084.1	P52799
187	NK052-1	EGFR	Pan-specific	Epidermal growth factor receptor-tyrosine kinase	NP_005219	P00533
188	PK010	EGFR	Y1172	Epidermal growth factor receptor-tyrosine kinase	NP_005219	P00533
189	PK010-2	EGFR	Y1172	Epidermal growth factor receptor-tyrosine kinase	NP_005219	P00533
190	PK011-1	EGFR	Y1197	Epidermal growth factor receptor-tyrosine kinase	NP_005219	P00533
191	PK121	EGFR	T693	Epidermal growth factor receptor-tyrosine kinase	NP_005219	P00533
192	PK122-1	EGFR	Y1092	Epidermal growth factor receptor-tyrosine kinase	NP_005219	P00533
193	PK123	EGFR	Y1110	Epidermal growth factor receptor-tyrosine kinase	NP_005219	P00533
194	NN038-1	eIF2a	Pan-specific	Eukaryotic translation initiation factor 2 alpha	NP_004085	P05198
195	PN028-1	eIF2a	S52	Eukaryotic translation initiation factor 2 alpha	NP_004085	P05198
196	PN028-2	eIF2a	S52	Eukaryotic translation initiation factor 2 alpha	NP_004085	P05198
197	PN172	eIF4B	S422	Eukaryotic translation initiation factor 4B	NP_001408.2	P23588
198	NN039-1	eIF4E	Pan-specific	Eukaryotic translation initiation factor 4 (mRNA cap binding protein)	NP_001959	P06730
199	PN030-1	eIF4E	S209	Eukaryotic translation initiation factor 4 (mRNA cap binding protein)	NP_001959	P06730
200	PN030-2	eIF4E	S209	Eukaryotic translation initiation factor 4 (mRNA cap binding protein)	NP_001959	P06730
201	PN031	eIF4G	S1107	Eukaryotic translation initiation factor 4 gamma 1	NP_004944	Q04637
202	PN193	eIF4G	S1232	Eukaryotic translation initiation factor 4 gamma 1	NP_004944	Q04637
203	NN168	Elk-1	Pan-specific	ETS domain-containing protein Elk-1	NP_001107595.1	P19419
204	PN149	Elk-1	S383	ETS domain-containing protein Elk-1	NP_001107595.1	P19419
205	PN170	Elk-1	S389	ETS domain-containing protein Elk-1	NP_001107595.1	P19419
206	NN173	Epcam	Pan-specific	Epithelial cell adhesion molecule	NP_002345.2	P16422
207	NK053	EphA1	Pan-specific	Ephrin type-A receptor 1 protein-tyrosine kinase	NP_005223	P21709
208	PN198	ER-alpha	S104	estrogen receptor alpha	NP_000116.2	P03372
209	NK054-1	ErbB2 (HER2)	Pan-specific	ErbB2 (Neu) receptor-tyrosine kinase	NP_004439	P04626
210	NK054-2	ErbB2 (HER2)	Pan-specific	ErbB2 (Neu) receptor-tyrosine kinase	NP_004439	P04626
211	PK013-1	ErbB2 (HER2)	Y1248	ErbB2 (Neu) receptor-tyrosine kinase	NP_004439	P04626
212	PK013-2	ErbB2 (HER2)	Y1248	ErbB2 (Neu) receptor-tyrosine kinase	NP_004439	P04626
213	PK134	ErbB2 (HER2)	T686	ErbB2 (Neu) receptor-tyrosine kinase	NP_004439	P04626
214	PK163	ErbB3	Y1328	Tyrosine kinase-type cell surface receptor HER3	NP_001005915.1	P21860
215	NK055-NK056	Erk1 (MAPK3)+ Erk2 (MAPK1)	Pan-specific	Extracellular regulated protein-serine kinase 1 (p44 MAP kinase)+Extracellular regulated protein-serine kinase 2 (p42 MAP kinase)	AAA36142.1, NP_002736	P27361
216	NK055-NK056-2	Erk1 (MAPK3)+ Erk2 (MAPK1)	Pan-specific	Extracellular regulated protein-serine kinase 1 (p44 MAP kinase)+Extracellular regulated protein-serine kinase 2 (p42 MAP kinase)	AAA36142.1, NP_002736	P27361
217	NK055-NK056-4	Erk1 (MAPK3)+ Erk2 (MAPK1)	Pan-specific	Extracellular regulated protein-serine kinase 1 (p44 MAP kinase)+Extracellular regulated protein-serine kinase 2 (p42 MAP kinase)	AAA36142.1, NP_002736	P27361
218	NK055-NK056-5	Erk1 (MAPK3)+ Erk2 (MAPK1)	Pan-specific	Extracellular regulated protein-serine kinase 1 (p44 MAP kinase)+Extracellular regulated protein-serine kinase 2 (p42 MAP kinase)	AAA36142.1, NP_002736	P27361
219	NK055-NK056-6	Erk1 (MAPK3)+ Erk2 (MAPK1)	Pan-specific	Extracellular regulated protein-serine kinase 1 (p44 MAP kinase)+Extracellular regulated protein-serine kinase 2 (p42 MAP kinase)	AAA36142.1, NP_002736	P27361
220	NK055-NK056-8	Erk1 (MAPK3)+ Erk2 (MAPK1)	Pan-specific	Extracellular regulated protein-serine kinase 1 (p44 MAP kinase)+Extracellular regulated protein-serine kinase 2 (p42 MAP kinase)	AAA36142.1, NP_002736	P27361
221	PK168-PK169	Erk1 (MAPK3)+ Erk2 (MAPK1)	Y204	Extracellular regulated protein-serine kinase 1 (p44 MAP kinase)+Extracellular regulated protein-serine kinase 2 (p42 MAP kinase)	AAA36142.1, NP_002736	P27361
222	PK170-PK171	Erk1 (MAPK3)+ Erk2 (MAPK1)	T202	Extracellular regulated protein-serine kinase 1 (p44 MAP kinase)+Extracellular regulated protein-serine kinase 2 (p42 MAP kinase)	AAA36142.1, NP_002736	P27361
223	NK056	Erk2 (MAPK1)	Pan-specific	Extracellular regulated protein-serine kinase 2 (p42 MAP kinase)	NP_002736	P28482
224	NK057-2	Erk3 (MAPK6)	Pan-specific	Extracellular regulated protein-serine kinase 3	NP_002739	Q16659
225	NK058	Erk4 (MAPK4)	Pan-specific	Extracellular regulated protein-serine kinase 4	NP_002738	P31152
226	NK206-1	Erk5 (MAPK7)	Pan-specific	Extracellular regulated protein-serine kinase 5 (Big MAP kinase 1 (BMK1))	NP_620602	Q13164
227	NK206-2	Erk5 (MAPK7)	Pan-specific	Extracellular regulated protein-serine kinase 5 (Big MAP kinase 1 (BMK1))	NP_620602	Q13164
228	NK206-3	Erk5 (MAPK7)	Pan-specific	Extracellular regulated protein-serine kinase 5 (Big MAP kinase 1 (BMK1))	NP_620602	Q13164
229	PK016	Erk5 (MAPK7)	T218+Y220	Extracellular regulated protein-serine kinase 5 (Big MAP kinase 1 (BMK1))	NP_620602	Q13164
230	PK016-3	Erk5 (MAPK7)	T218+Y220	Extracellular regulated protein-serine kinase 5 (Big MAP kinase 1 (BMK1))	NP_620602	Q13164
231	NN040	ERP57	Pan-specific	ER protein 57 kDa (protein disulfide isomerase-associated 3; 58 kDa glucose regulated protein)	NP_005304	P30101
232	NN041	ERP72	Pan-specific	ER protein 72 kDa (protein disulfide isomerase-associated 4)	NP_004902	P13667
233	PN174	Ezrin	T567	cytovillin 2	NP_001104547.1	P15311
234	PN175	Ezrin	Y353	cytovillin 2	NP_001104547.1	P15311
235	NK060	FAK	Pan-specific	Focal adhesion protein-tyrosine kinase	NP_005598	Q05397
236	PK017	FAK	Y397	Focal adhesion protein-tyrosine kinase	NP_005598	Q05397
237	PK017-1	FAK	Y397	Focal adhesion protein-tyrosine kinase	NP_005598	Q05397
238	PK018-3	FAK	Y576	Focal adhesion protein-tyrosine kinase	NP_005598	Q05397
239	PK020	FAK	S722	Focal adhesion protein-tyrosine kinase	NP_005598	Q05397
240	PK020-3	FAK	S722	Focal adhesion protein-tyrosine kinase	NP_005598	Q05397
241	PK021	FAK	S732	Focal adhesion protein-tyrosine kinase	NP_005598	Q05397
242	PK022-2	FAK	S843	Focal adhesion protein-tyrosine kinase	NP_005598	Q05397
243	PK024	FAK	S910	Focal adhesion protein-tyrosine kinase	NP_005598	Q05397

244	PK151	FAK	Y576/Y577	Focal adhesion protein-tyrosine kinase	NP_005598	Q05397
245	NN042	FAS	Pan-specific	Tumor necrosis factor superfamily member 6 (Apo1, CD95)	NP_003789	P25445
246	NN043	FasL	Pan-specific	Tumor necrosis factor ligand, member 6	NP_000630	P48023
247	NK061	Fes	Pan-specific	Fes/Fps protein-tyrosine kinase	NP_001996	P07332
248	NN172	FHL2	Pan-specific	Four and a half LIM domains protein 2	NP_001034581.1	Q14192
249	NN127	FKBP52	Pan-specific	FK506-binding protein 4	NP_002005	Q02790
250	PN194	FKHR	S256	Forkhead box protein O1	NP_002006.2	Q12778
251	PN195	FKHR	S319	Forkhead box protein O1	NP_002006.2	Q12778
252	PN145-PN032	FKHRL1	T32	Forkhead-like transcription factor 1 (FOXO3A)	NP_001446	O43524
253	PN145-PN032-PN153	FKHRL1	T32	Forkhead-like transcription factor 1 (FOXO3A)	NP_001446	O43524
254	NN044	Fos	Pan-specific	Fos-c FBJ murine osteosarcoma oncoprotein-related transcription factor	NP_005243	P01100
255	PN033	Fos	T232	Fos-c FBJ murine osteosarcoma oncoprotein-related transcription factor	NP_005243	P01100
256	PN146	FRS2	Y348	Fibroblast growth factor receptor substrate 2	NP_001036020.1	Q8WU20
257	NK065	Fyn	Pan-specific	Fyn proto-oncogene-encoded protein-tyrosine kinase	NP_002028	P06241
258	PN192	Gab1	Y627	GRB2-associated binder 1	NP_002030.2	Q13480
259	PN098	GAP-43	S41	Growth associated protein 43 (Neuromodulin)	NP_002036	P17677
260	PN196	GATA1	S142	Erythroid transcription factor	NP_002040.1	P15976
261	NK066	GCK	Pan-specific	Germinal centre protein-serine kinase	NP_004570	Q12851
262	PN034	GFAP	S8	Glial fibrillary acidic protein	NP_002046	P14136
263	PN178	GluR1	S849	Glutamate receptor 1	NP_000818.2	P42261
264	NN045	GNB2L1	Pan-specific	Guanine nucleotide-binding protein beta (receptor for activated C kinase 1 (RACK1))	NP_006089	P63244
265	NK067	GRK2 (BARK1)	Pan-specific	G protein-coupled receptor-serine kinase 2	NP_001610	P25098
266	PK025	GRK2 (BARK1)	S670	G protein-coupled receptor-serine kinase 2	NP_001610	P25098
267	NK068	GRK3 (BARK2)	Pan-specific	G protein-coupled receptor-serine kinase 3	NP_005151	P35626
268	NN046	GroEL	Pan-specific	GroEL homolog (may correspond to Hsp60)	NP_002147	P10809
269	NN047	Grp75	Pan-specific	Glucose regulated protein 75	NP_004125	P38646
270	NN048	Grp78	Pan-specific	Glucose regulated protein 78	NP_005338	P11021
271	NN048-2	Grp78	Pan-specific	Glucose regulated protein 78	NP_005338	P11021
272	NN049	Grp94	Pan-specific	Glucose regulated protein 94 (endoplasmin)	NP_003290	P14625
273	NK069-NK070	GSK3a	Pan-specific	Glycogen synthase-serine kinase 3 alpha	NP_063937	P49840
274	NK069-NK070-2	GSK3a	Pan-specific	Glycogen synthase-serine kinase 3 alpha	NP_063937	P49840
275	PK026-PK027-1	GSK3a	S21	Glycogen synthase-serine kinase 3 alpha	NP_063937	P49840
276	PK028-PK029-1	GSK3a	Y279	Glycogen synthase-serine kinase 3 alpha	NP_063937	P49840
277	NK070	GSK3b	Pan-specific	Glycogen synthase-serine kinase 3 beta	NP_002084	P49841
278	NK070-1	GSK3b	Pan-specific	Glycogen synthase-serine kinase 3 beta	NP_002084	P49841
279	NK071	Haspin	Pan-specific	Haploid germ cell-specific nuclear protein-serine kinase	NP_114171	Q8TF76
280	NN169	HDAC4	Pan-specific	Histone deacetylase 4	NP_006028.2	P56524
281	PN179-PN180-PN181	HDAC4	S246	Histone deacetylase 4	NP_006028.2	P56524
282	PN188	HDAC5	S498	Histone deacetylase 5	NP_001015053.1	Q9UQL6
283	NN050	hHR23B	Pan-specific	UV excison repair protein RAD23 homolog B	NP_002865	P54727
284	NN051	Hip	Pan-specific	Hsp70/Hsc70 interacting protein (ST13)	NP_003923	P50502
285	PN035	Histone H1	phospho CDK1 sites	Histone H1 phosphorylated	NP_005316	Q02539
286	PN036	Histone H2A.X	S140	Histone H2A variant X	NP_002096	P16104
287	PN037	Histone H2B	S15	Histone H2B	NP_778225	P33778
288	PN038	Histone H3	S11	Histone H3.3	NP_003521	P84243
289	PN039	Histone H3	S29	Histone H3.3	NP_003521	P84243
290	PN100	Histone H3	T12	Histone H3.3	NP_003521	P84243
291	PN101	Histone H3	T4	Histone H3.3	NP_003521	P84243
292	PN101-2	Histone H3	T4	Histone H3.3	NP_003521	P84243
293	NN052	HO1	Pan-specific	Heme oxygenase 1	NP_002124	P09601
294	NN053	HO2	Pan-specific	Heme oxygenase 2	NP_002125	P30519
295	NK072	Hpk1 (MAP4K1)	Pan-specific	Hematopoetic progenitor protein-serine kinase 1	NP_009112	Q92918
296	NN054	Hsc70	Pan-specific	Heat shock 70 kDa protein 8	NP_006588	P11142
297	NN054-2	Hsc70	Pan-specific	Heat shock 70 kDa protein 8	NP_006588	P11142
298	NN055	HSF4	Pan-specific	Heat shock transcription factor 4	NP_001529	Q9ULV5
299	NN062	Hsp105	Pan-specific	Heat shock 105 kDa protein	NP_006635	Q92598
300	NN152-1	Hsp27	Pan-specific	Heat shock 27 kDa protein beta 1 (HspB1)	NP_001531	P04792
301	PN040-1	Hsp27	S15	Heat shock 27 kDa protein beta 1 (HspB1)	NP_001531	P04792
302	PN040-2	Hsp27	S15	Heat shock 27 kDa protein beta 1 (HspB1)	NP_001531	P04792
303	PN041	Hsp27	S78	Heat shock 27 kDa protein beta 1 (HspB1)	NP_001531	P04792
304	PN042-1	Hsp27	S82	Heat shock 27 kDa protein beta 1 (HspB1)	NP_001531	P04792

305	PN042-2	Hsp27	S82	Heat shock 27 kDa protein beta 1 (HspB1)	NP_001531	P04792
306	PN042-3	Hsp27	S82	Heat shock 27 kDa protein beta 1 (HspB1)	NP_001531	P04792
307	NN057	Hsp40	Pan-specific	DnaJ homolog, subfamily B member 1	NP_006136	P25685
308	NN057-2	Hsp40	Pan-specific	DnaJ homolog, subfamily B member 1	NP_006136	P25685
309	NN057-3	Hsp40	Pan-specific	DnaJ homolog, subfamily B member 1	NP_006136	P25685
310	NN058	Hsp47	Pan-specific	Heat shock 47 kDa protein (collagen-binding protein 1, colligin 1)	NP_001226	P29043
311	NN059-1	Hsp60	Pan-specific	Heat shock 60 kDa protein 1 (chaperonin, CPN60)	NP_002147	P10809
312	NN059-2	Hsp60	Pan-specific	Heat shock 60 kDa protein 1 (chaperonin, CPN60)	NP_002147	P10809
313	NN059-3	Hsp60	Pan-specific	Heat shock 60 kDa protein 1 (chaperonin, CPN60)	NP_002147	P10809
314	NN060	Hsp70	Pan-specific	Heat shock 70 kDa protein 1	NP_005336	P08107
315	NN060-2	Hsp70	Pan-specific	Heat shock 70 kDa protein 1	NP_005336	P08107
316	NN060-3	Hsp70	Pan-specific	Heat shock 70 kDa protein 1	NP_005336	P08107
317	NN164	Hsp90a	Pan-specific	Heat shock 90 kDa protein alpha	NP_005339	P07900
318	NN061	Hsp90a/b	Pan-specific	Heat shock 90 kDa protein alpha/beta	NP_005339	P07900
319	NN061-16	Hsp90a/b	Pan-specific	Heat shock 90 kDa protein alpha/beta	NP_005339	P07900
320	NN061-2	Hsp90a/b	Pan-specific	Heat shock 90 kDa protein alpha/beta	NP_005339	P07900
321	NN061-3	Hsp90a/b	Pan-specific	Heat shock 90 kDa protein alpha/beta	NP_005339	P07900
322	NN061-4	Hsp90a/b	Pan-specific	Heat shock 90 kDa protein alpha/beta	NP_005339	P07900
323	NN165	Hsp90b	Pan-specific	Heat shock 90 kDa protein beta	NP_031381	P08238
324	NN165-1	Hsp90b	Pan-specific	Heat shock 90 kDa protein beta	NP_031381	P08238
325	PN176	Hsp90b	S255	Heat shock 90 kDa protein beta	NP_031381	P08238
326	NN063	HspBP1	Pan-specific	Hsp70 binding protein 1	NP_036399	Q9NZL4
327	PN103	Huntingtin	S421	Huntington's disease protein	NP_002102	P42858
328	NN130	I1PP2A	Pan-specific	Acidic leucine-rich nuclear phosphoprotein 32 family member A	NP_006296	P39687
329	NN131	I2PP2A	Pan-specific	Protein SET	NP_003002	Q01105
330	NK073	ICK	Pan-specific	Intestinal cell protein-serine kinase (MAK-related kinase (MRK)	NP_057597	Q9UPZ9
331	NK074	IGF1R	Pan-specific	Insulin-like growth factor 1 receptor protein-tyrosine kinase	NP_000866	P08069
332	PK152	IGF1R	Y1280	Insulin-like growth factor 1 receptor protein-tyrosine kinase	NP_000866	P08069
333	PK153	IGF1R	Y1165/Y1166	Insulin-like growth factor 1 receptor protein-tyrosine kinase	NP_000866	P08069
334	PK139	IGF1Rb/IRb	Y1161/Y1185	Insulin-like growth factor 1 /Insulin receptorreceptor beta	NP_000866	P08069
335	NN064	IkBa	Pan-specific	Inhibitor of NF-kappa-B alpha (MAD3)	NP_065390	P25963
336	NN064-2	IkBa	Pan-specific	Inhibitor of NF-kappa-B alpha (MAD3)	NP_065390	P25963
337	NN064-4	IkBa	Pan-specific	Inhibitor of NF-kappa-B alpha (MAD3)	NP_065390	P25963
338	PN164	IkBa	Y42	Inhibitor of NF-kappa-B alpha (MAD3)	NP_065390	P25963
339	NN065	IkBb	Pan-specific	Inhibitor of NF-kappa-B beta (thyroid receptor interacting protein 9)	NP_002494	Q15653
340	PN168	IkBe	S22	NF-kappa-B inhibitor epsilon	NP_004547.2	O00221
341	NK075-1	IKKa	Pan-specific	Inhibitor of NF-kappa-B protein-serine kinase alpha (CHUK)	NP_001269	O15111
342	NK075-2	IKKa	Pan-specific	Inhibitor of NF-kappa-B protein-serine kinase alpha (CHUK)	NP_001269	O15111
343	NK075-3	IKKa	Pan-specific	Inhibitor of NF-kappa-B protein-serine kinase alpha (CHUK)	NP_001269	O15111
344	NK075-4	IKKa	Pan-specific	Inhibitor of NF-kappa-B protein-serine kinase alpha (CHUK)	NP_001269	O15111
345	NK075-5	IKKa	Pan-specific	Inhibitor of NF-kappa-B protein-serine kinase alpha (CHUK)	NP_001269	O15111
346	NK075-6	IKKa	Pan-specific	Inhibitor of NF-kappa-B protein-serine kinase alpha (CHUK)	NP_001269	O15111
347	PK030-PK031	IKKa	S180	Inhibitor of NF-kappa-B protein-serine kinase alpha (CHUK)	NP_001269	O15111
348	PK154	IKKa	T23	Inhibitor of NF-kappa-B protein-serine kinase alpha (CHUK)	NP_001269	O15111
349	NK076-1	IKKb	Pan-specific	Inhibitor of NF-kappa-B protein-serine kinase beta	NP_001547	O14920
350	NK076-2	IKKb	Pan-specific	Inhibitor of NF-kappa-B protein-serine kinase beta	NP_001547	O14920
351	NK076-3	IKKb	Pan-specific	Inhibitor of NF-kappa-B protein-serine kinase beta	NP_001547	O14920
352	NK076-4	IKKb	Pan-specific	Inhibitor of NF-kappa-B protein-serine kinase beta	NP_001547	O14920
353	NN161	IKKg (NEMO)	Pan-specific	I-kappa-B kinase gamma/NF-kappa-B essential modulator	NP_003630	Q9Y6K9
354	NK078-2	ILK1	Pan-specific	Integrin-linked protein-serine kinase 1	NP_034692	Q13418
355	NK078-3	ILK1	Pan-specific	Integrin-linked protein-serine kinase 1	NP_034692	Q13418
356	PN043	Integrin a4	S1027	Integrin alpha 4 (VLA4)	NP_000876	P13612
357	PN044	Integrin b1	S785	Integrin beta 1 (fibronectin receptor beta subunit, CD29 antigen)	NP_002202	P05556
358	NK079	IR (INSR)	Pan-specific	Insulin receptor beta chain	NP_000199	P06213
359	PK032-1	IR (INSR)	Y999	Insulin receptor	NP_000199	P06213
360	PK033	IR/IGF1R (INSR)	Y1189/Y1190	Insulin receptor / Insulin-like growth factor 1 receptor	NP_000866	P06213
361	NK080	IRAK1	Pan-specific	Interleukin 1 receptor-associated kinase 1 (Pelle-like protein kinase)	NP_001560	P51617
362	NK080-2	IRAK1	Pan-specific	Interleukin 1 receptor-associated kinase 1 (Pelle-like protein kinase)	NP_001560	P51617
363	NK081	IRAK2	Pan-specific	Interleukin 1 receptor-associated kinase 2	NP_001561	O43187
364	NK081-2	IRAK2	Pan-specific	Interleukin 1 receptor-associated kinase 2	NP_001561	O43187
365	NK082	IRAK3	Pan-specific	Interleukin 1 receptor-associated kinase 3	NP_009130	Q9Y616

366	NK083-1	IRAK4	Pan-specific	Interleukin 1 receptor-associated kinase 4	NP_057207	Q9NWZ3
367	NK083-2	IRAK4	Pan-specific	Interleukin 1 receptor-associated kinase 4	NP_057207	Q9NWZ3
368	PN045	IRS1	Y612	Insulin receptor substrate 1	NP_005535	P35568
369	PN046-2	IRS1	Y1179	Insulin receptor substrate 1	NP_005535	P35568
370	PN117	IRS1	S312	Insulin receptor substrate 1	NP_005535	P35568
371	PN118	IRS1	S639	Insulin receptor substrate 1	NP_005535	P35568
372	NK084-1	JAK1	Pan-specific	Janus protein-tyrosine kinase 1	NP_002218	P23458
373	NK084-2	JAK1	Pan-specific	Janus protein-tyrosine kinase 1	NP_002218	P23458
374	PK126	JAK1	Y1034	Janus protein-tyrosine kinase 1	NP_002218	P23458
375	NK085	JAK2	Pan-specific	Janus protein-tyrosine kinase 2	NP_004963	O60674
376	PK034-1	JAK2	Y1007+Y1008	Janus protein-tyrosine kinase 2	NP_004963	O60674
377	PK034-2	JAK2	Y1007+Y1008	Janus protein-tyrosine kinase 2	NP_004963	O60674
378	NK086	JAK3	Pan-specific	Janus protein-tyrosine kinase 3	NP_000206	P52333
379	NK087	TAO3 (JIK)	Pan-specific	STE20-like protein-serine kinase	NP_057365	Q9H2K8
380	NK217	JNK1 (MAPK8)	Pan-specific	Jun N-terminus protein-serine kinase (stress-activated protein kinase (SAPK)) 1	NP_620637.1	P45983
381	NK088-1	JNK1/2/3	Pan-specific	Jun N-terminus protein-serine kinase (stress-activated protein kinase (SAPK)) 1/2/3	NP_002741	P45983
382	NK088-2	JNK1/2/3	Pan-specific	Jun N-terminus protein-serine kinase (stress-activated protein kinase (SAPK)) 1/2/3	NP_002741	P45983
383	NK088-3	JNK1/2/3	Pan-specific	Jun N-terminus protein-serine kinase (stress-activated protein kinase (SAPK)) 1/2/3	NP_002741	P45983
384	PK035-1	JNK1/2/3	T183 + Y185	Jun N-terminus protein-serine kinase (stress-activated protein kinase (SAPK)) 1/2/3	NP_002741	P45983
385	PK035-2	JNK1/2/3	T183 + Y185	Jun N-terminus protein-serine kinase (stress-activated protein kinase (SAPK)) 1/2/3	NP_002741	P45983
386	PK035-4	JNK1/2/3	T183 + Y185	Jun N-terminus protein-serine kinase (stress-activated protein kinase (SAPK)) 1/2/3	NP_002741	P45983
387	NK189	JNK2 (MAPK9)	Pan-specific	Jun N-terminus protein-serine kinase (stress-activated protein kinase (SAPK)) 2	NP_002744	P45984
388	NK196	JNK2/3	Pan-specific	Jun N-terminus protein-serine kinase (stress-activated protein kinase (SAPKa/b)) 2/3	NP_002743.3	P45984
389	NK197	JNK3 (MAPK10)	Pan-specific	Jun N-terminus protein-serine kinase (stress-activated protein kinase (SAPKb)) 3	NP_002744.1	P53779
390	NN162	Jun	Pan-specific	Jun proto-oncogene-encoded AP1 transcription factor	NP_002219	P05412
391	PN047	Jun	S63	Jun proto-oncogene-encoded AP1 transcription factor	NP_002219	P05412
392	PN048-1	Jun	S73	Jun proto-oncogene-encoded AP1 transcription factor	NP_002219	P05412
393	PN048-2	Jun	S73	Jun proto-oncogene-encoded AP1 transcription factor	NP_002219	P05412
394	PN154	Jun	S243	Jun proto-oncogene-encoded AP1 transcription factor	NP_002219	P05412
395	PN155	Jun	Y170	Jun proto-oncogene-encoded AP1 transcription factor	NP_002219	P05412
396	PN163	Jun	T91	Jun proto-oncogene-encoded AP1 transcription factor	NP_002219	P05412
397	NP004	KAP	Pan-specific	Cyclin-dependent kinase associated phosphatase (CDK inhibitor 3, CIP2)	NP_005183	Q16667
398	NN153	KDEL receptor 1	Pan-specific	ER lumen protein retaining receptor 1	NP_006792.1	P24390
399	NK089	KHS (MAP4K5)	Pan-specific	Kinase homologous to SPS1/STE20 (MAP kinase kinase kinase protein-serine kinase 5 (MEKKK5)	NP_006566	Q9Y4K4
400	PK036	Kit	Y703	Kit/Steel factor receptor-tyrosine kinase	NP_006566	P10721
401	PK037	Kit	Y730	Kit/Steel factor receptor-tyrosine kinase	NP_006566	P10721
402	PK038	Kit	Y936	Kit/Steel factor receptor-tyrosine kinase	NP_006566	P10721
403	PK150	Kit	Y721	Kit/Steel factor receptor-tyrosine kinase	NP_006566	P10721
404	NK090	Ksr1	Pan-specific	Protein-serine kinase suppressor of Ras 1	NP_055053.1	Q8IVT5
405	NK090-2	Ksr1	Pan-specific	Protein-serine kinase suppressor of Ras 1	NP_055053.1	Q8IVT5
406	NP005	LAR	Pan-specific	LCA antigen-related (LAR) receptor tyrosine phosphatase	NP_002831	P10586
407	NK091	LATS1	Pan-specific	Large tumor suppressor 1 protein-serine kinase (WARTS)	NP_004681	O95835
408	NK092-2	Lck	Pan-specific	Lymphocyte-specific protein-tyrosine kinase	NP_005347	P06239
409	NK092-3	Lck	Pan-specific	Lymphocyte-specific protein-tyrosine kinase	NP_005347	P06239
410	PK039	Lck	S158	Lymphocyte-specific protein-tyrosine kinase	NP_005347	P06239
411	PK040	Lck	Y192	Lymphocyte-specific protein-tyrosine kinase	NP_005347	P06239
412	PK041	Lck	Y505	Lymphocyte-specific protein-tyrosine kinase	NP_005347	P06239
413	PK149	Lck	Y394	Lymphocyte-specific protein-tyrosine kinase	NP_005347	P06239
414	NK093	LIMK1	Pan-specific	LIM domain kinase 1	NP_002305	P53667
415	PK042-PK144	LIMK1	Y507+T508	LIM domain kinase 1	NP_002305	P53667
416	NK095	Lyn	Pan-specific	Yes-related protein-tyrosine kinase	NP_002341	P07948
417	PK043	Lyn	Y508	Yes-related protein-tyrosine kinase	NP_002341	P07948
418	NK096	MAK	Pan-specific	Male germ cell-associated protein-serine kinase	NP_005897	P20794
419	NK097	MAPKAPK2	Pan-specific	Mitogen-activated protein kinase-activated protein kinase 2	NP_116584	P49137
420	PK044	MAPKAPK2	T222	Mitogen-activated protein kinase-activated protein kinase 2	NP_004750	P49137
421	PN049-PN112-1	MAPKAPK2a	T334	Mitogen-activated protein kinase-activated protein kinase 2 alpha	NP_004750	P49137
422	PN049-PN112-2	MAPKAPK2a	T334	Mitogen-activated protein kinase-activated protein kinase 2 alpha	NP_004750	P49137
423	PN050-1	MARCKS	S159+S163	Myristoylated alanine-rich protein kinase C substrate	NP_002347	P29966
424	NK098	MARK	Pan-specific	MAP/microtubule affinity-regulating protein-serine kinase 1	NP_061120	Q9P0L2
425	NN067	Mcl1	Pan-specific	Myeloid cell leukemia differentiation protein 1	NP_068779	Q07820
426	PN169	MDM2	S166	double minute 2	NP_002383.2	Q00987

427	NN155	MEF-2	Pan-specific	Myelin expression factor 2 (MYEF2)	NP_057216.2	Q9P2K5
428	NK099-1	MEK1 (MAP2K1)	Pan-specific	MAPK/ERK protein-serine kinase 1 (MKK1)	NP_002746	Q02750
429	NK099-2	MEK1 (MAP2K1)	Pan-specific	MAPK/ERK protein-serine kinase 1 (MKK1)	NP_002746	Q02750
430	NK099-3	MEK1 (MAP2K1)	Pan-specific	MAPK/ERK protein-serine kinase 1 (MKK1)	NP_002746	Q02750
431	NK099-4	MEK1 (MAP2K1)	Pan-specific	MAPK/ERK protein-serine kinase 1 (MKK1)	NP_002746	Q02750
432	NK099-5	MEK1 (MAP2K1)	Pan-specific	MAPK/ERK protein-serine kinase 1 (MKK1)	NP_002746	Q02750
433	PK046-1	MEK1 (MAP2K1)	T292	MAPK/ERK protein-serine kinase 1 (MKK1)	NP_002746	Q02750
434	PK046-2	MEK1 (MAP2K1)	T292	MAPK/ERK protein-serine kinase 1 (MKK1)	NP_002746	Q02750
435	PK046-3	MEK1 (MAP2K1)	T292	MAPK/ERK protein-serine kinase 1 (MKK1)	NP_002746	Q02750
436	PK047-2	MEK1 (MAP2K1)	S298	MAPK/ERK protein-serine kinase 1 (MKK1)	NP_002746	Q02750
437	PK048-1	MEK1 (MAP2K1)	T386	MAPK/ERK protein-serine kinase 1 (MKK1)	NP_002746	Q02750
438	PK048-2	MEK1 (MAP2K1)	T386	MAPK/ERK protein-serine kinase 1 (MKK1)	NP_002746	Q02750
439	PK048-3	MEK1 (MAP2K1)	T386	MAPK/ERK protein-serine kinase 1 (MKK1)	NP_002746	Q02750
440	PK045-PN007	MEK1/2 (MAP2K1/2)	S218+S222	MAPK/ERK protein-serine kinase 1/2 (MKK1/2)	NP_002746	Q02750
441	NK221	MEK1/2 (MAP2K1/2)	Pan-specific	MAPK/ERK protein-serine kinase 1/2 (MKK1/2)	NP_002746	Q02750
442	NK100-1	MEK2 (MAP2K2)	Pan-specific	MAPK/ERK protein-serine kinase 2 (MKK2)	AAH00471.1	P36507
443	NK100-2	MEK2 (MAP2K2)	Pan-specific	MAPK/ERK protein-serine kinase 2 (MKK2)	AAH00471.1	P36507
444	NK100-3	MEK2 (MAP2K2)	Pan-specific	MAPK/ERK protein-serine kinase 2 (MKK2)	AAH00471.1	P36507
445	PK049	MEK2 (MAP2K2)	T394	MAPK/ERK protein-serine kinase 2 (MKK2)	AAH00471.1	P36507
446	PK049-2	MEK2 (MAP2K2)	T394	MAPK/ERK protein-serine kinase 2 (MKK2)	AAH00471.1	P36507
447	PK050	MEK2 (MAP2K2)	T394	MAPK/ERK protein-serine kinase 2 (MKK2) (mouse)	NP_075627	P36507
448	NK101	MEK3 (MAP2K3)	Pan-specific	MAPK/ERK protein-serine kinase 3 (MKK3)	NP_659732	P46734
449	NK101-3	MEK3 (MAP2K3)	Pan-specific	MAPK/ERK protein-serine kinase 3 (MKK3)	NP_659732	P46734
450	PK127	MEK3 (MAP2K3)	S218	MAPK/ERK protein-serine kinase 3 (MKK3)	NP_659732	P46734
451	PK051	MEK3/6 (MAP2K3/6)	S218/S207	MAPK/ERK protein-serine kinase 3/6 (MKK3/6)	NP_002747	P46734
452	PK051-2	MEK3/6 (MAP2K3/6)	S218/S207	MAPK/ERK protein-serine kinase 3/6 (MKK3/6)	NP_002747	P46734
453	PK051-3	MEK3/6 (MAP2K3/6)	S218/S207	MAPK/ERK protein-serine kinase 3/6 (MKK3/6)	NP_002747	P46734
454	NK102	MEK3b (MAP2K3)	Pan-specific	MAPK/ERK protein-serine kinase 3 beta isoform (MKK3 beta)	NP_659731	P46734
455	NK103	MEK4 (MAP2K4)	Pan-specific	MAPK/ERK protein-serine kinase 4 (MKK4)	NP_003001	P45985
456	NK103-2	MEK4 (MAP2K4)	Pan-specific	MAPK/ERK protein-serine kinase 4 (MKK4)	NP_003001	P45985
457	NK103-3	MEK4 (MAP2K4)	Pan-specific	MAPK/ERK protein-serine kinase 4 (MKK4)	NP_003001	P45985
458	PK052	MEK4 (MAP2K4)	S257+T261	MAPK/ERK protein-serine kinase 4 (MKK4)	NP_003001	P45985
459	PK155	MEK5 (MAP2K5)	S311+T315	MAPK/ERK protein-serine kinase 5 (MKK5)	NP_660143	Q13163
460	NK104	MEK5 (MAP2K5)	Pan-specific	MAPK/ERK protein-serine kinase 5 (MKK5)	NP_660143	Q13163
461	NK104-2	MEK5 (MAP2K5)	Pan-specific	MAPK/ERK protein-serine kinase 5 (MKK5)	NP_660143	Q13163
462	NK105-1	MEK6 (MAP2K6)	Pan-specific	MAPK/ERK protein-serine kinase 6 (MKK6)	NP_002749	P52564
463	PK129	MEK6 (MAP2K6)	S207	MAPK/ERK protein-serine kinase 6 (MKK6)	NP_002749	P52564
464	NK106-2	MEK7 (MAP2K7)	Pan-specific	MAPK/ERK protein-serine kinase 7 (MKK7)	NP_005034	O14733
465	NK107	MEKK1 (MAP3K1)	Pan-specific	MAPK/ERK kinase kinase 1	NP_005912.1	Q13233
466	NK107-2	MEKK1 (MAP3K1)	Pan-specific	MAPK/ERK kinase kinase 1	NP_005912.1	Q13233
467	NK107-3	MEKK1 (MAP3K1)	Pan-specific	MAPK/ERK kinase kinase 1	NP_005912.1	Q13233
468	NK107-4	MEKK1 (MAP3K1)	Pan-specific	MAPK/ERK kinase kinase 1	NP_005912.1	Q13233
469	NK108	MEKK2 (MAP3K2)	Pan-specific	MAPK/ERK kinase kinase 2	NP_006600.3	Q9Y2U5
470	NK108-2	MEKK2 (MAP3K2)	Pan-specific	MAPK/ERK kinase kinase 2	NP_006600.3	Q9Y2U5
471	NK109	MEKK4 (MAP3K4)	Pan-specific	MAPK/ERK kinase kinase 4	NP_005913	Q9Y6R4
472	PK055-1	Met	Y1230+Y1234+Y1235	Hepatocyte growth factor (HGF) receptor-tyrosine kinase	NP_000236.	P08581
473	NP006	MKP1	Pan-specific	MAP kinase phosphatase 1 (CL100, VH1, DUSP1)	NP_004408	P28562
474	NP007	MKP2	Pan-specific	MAP kinase phosphatase 2 (VH2)	NP_001385	Q13115
475	PN051-1	MLC(MLRC2)	S19	Myosin regulatory light chain 2, smooth muscle isoform	NP_291024	P19105
476	PN051-2	MLC(MLRC2)	S19	Myosin regulatory light chain 2, smooth muscle isoform	NP_291024	P19105
477	NK208	MLK3	Pan-specific	Mixed-lineage protein-serine kinase 3	NP_002410	Q16584
478	PK056	MLK3	T277+S281	Mixed-lineage protein-serine kinase 3	NP_002410	Q16584
479	NN132	mMOB1	Pan-specific	Preimplantation protein 3	NP_056202	Q9Y3A3
480	PK057	Mnk1	T250+T255	MAP kinase-interacting protein-serine kinase 1 (calmodulin-activated)	NP_003675	Q9BUB5
481	NK111	Mnk2	Pan-specific	MAP kinase-interacting protein-serine kinase 2 (calmodulin-activated)	NP_060042	Q9HBH9
482	NN069	MSH2	Pan-specific	DNA mismatch repair protein mutS homolog2, colon cancer, nonpolyposis type 1	NP_000242	P43246
483	PK058	Msk1	S376	Mitogen & stress-activated protein-serine kinase 1	NP_004746	O75582
484	NK113-1	MST1	Pan-specific	Mammalian STE20-like protein-serine kinase 1 (KRS2)	NP_006273	Q13043
485	NK113-2	MST1	Pan-specific	Mammalian STE20-like protein-serine kinase 1 (KRS2)	NP_006273	Q13043
486	NK113-3	MST1	Pan-specific	Mammalian STE20-like protein-serine kinase 1 (KRS2)	NP_006273	Q13043
487	NK113-4	MST1	Pan-specific	Mammalian STE20-like protein-serine kinase 1 (KRS2)	NP_006273	Q13043

488	NK114	MST2	Pan-specific	Mammalian STE20-like protein-serine kinase 2 (KRS1)	NP_006272	Q13188
489	NK115	MST3	Pan-specific	Mammalian STE20-like protein-serine kinase 3	NP_003567	Q9Y6E0
490	PK116	mTOR (FRAP)	S2448	Mammalian target of rapamycin (FRAP)	NP_004949	P42345
491	PN186	Myc	S373	Myc proto-oncogene protein	NP_002458.2	P01106
492	PN199	Myc	T58	Myc proto-oncogene protein	NP_002458.2	P01106
493	PN182	MyoD	S200	Myoblast determination protein 1	NP_002469.2	P15172
494	PN052	MYPT1	T696	Myosin phosphatase target 1	NP_446342	O14974
495	PN187	NBS1	S343	Nijmegen breakage syndrome protein 1	NP_002476.2	O60934
496	NK117-1	Nek2	Pan-specific	NIMA (never-in-mitosis)-related protein-serine kinase 2	NP_002488	P51955
497	NK117-2	Nek2	Pan-specific	NIMA (never-in-mitosis)-related protein-serine kinase 2	NP_002488	P51955
498	NK117-3	Nek2	Pan-specific	NIMA (never-in-mitosis)-related protein-serine kinase 2	NP_002488	P51955
499	NK117-4	Nek2	Pan-specific	NIMA (never-in-mitosis)-related protein-serine kinase 2	NP_002488	P51955
500	NK117-5	Nek2	Pan-specific	NIMA (never-in-mitosis)-related protein-serine kinase 2	NP_002488	P51955
501	NK118	Nek4	Pan-specific	NIMA (never-in-mitosis)-related protein-serine kinase 4	NP_003148	P51957
502	NK119	Nek7	Pan-specific	NIMA (never-in-mitosis)-related protein-serine kinase 7	NP_598001	Q8TDX7
503	NN070	NFkappaB p50	Pan-specific	NF-kappa-B p50 nuclear transcription factor	NP_003989	P19838
504	NN071	NFkappaB p65	Pan-specific	NF-kappa-B p65 nuclear transcription factor	NP_003989	Q04206
505	PN053-1	NFkappaB p65	S276	NF-kappa-B p65 nuclear transcription factor	NP_003989	Q04206
506	PN156	NFkappaB p65	S529	NF-kappa-B p65 nuclear transcription factor	NP_003989	Q04206
507	PN157	NFkappaB p65	S536	NF-kappa-B p65 nuclear transcription factor	NP_003989	Q04206
508	NK207	NIK (MAP3K14)	Pan-specific	NF-kappa beta-inducing kinase	NP_003945.2	Q99558
509	NK212	NLK	Pan-specific	Serine/threonine protein kinase NLK	NP_057315.3	Q9UBE8
510	PN054	NMDAR2B	Y1474	N-methyl-D-aspartate (NMDA) glutamate receptor 2B subunit	NP_000825	Q13224
511	NN074	NME7	Pan-specific	Nucleotide diphosphate kinase 7 (nm23-H7)	NP_037462	Q9Y5B8
512	PN055-1	NR1	S896	N-methyl-D-aspartate (NMDA) glutamate receptor 1 subunit zeta	NP_000823	Q05586
513	NN075	NT5E	Pan-specific	Ecto-5'-nucleotidase (CD73 antigen)	NP_002517	P21589
514	NN083	p107	Pan-specific	Retinoblastoma (Rb) protein-related p107 (PRB1)	NP_002886.2	P28749
515	NN077	p18 INK4c	Pan-specific	p18 INK4c cyclin-dependent kinase inhibitor	NP_523240	P42773
516	NN078	p21 CDKI1	Pan-specific	cyclin-dependent kinase inhibitor 1 (MDA6)	NP_000380	P38936
517	NN081-NN120	p25	Pan-specific	CDK5 regulatory subunit p25	NP_003876.1	Q15078
518	NN080	p27 Kip1	Pan-specific	p27 cyclin-dependent kinase inhibitor 1B	NP_004055	P46527
519	PN056	p27 Kip1	T187	p27 cyclin-dependent kinase inhibitor 1B	NP_004055	P46527
520	PK060-1	p38a MAPK	T180+Y182	Mitogen-activated protein-serine kinase p38 alpha	NP_001306	Q16539
521	PK060-2	p38a MAPK	T180+Y182	Mitogen-activated protein-serine kinase p38 alpha	NP_001306	Q16539
522	PK060-3	p38a MAPK	T180+Y182	Mitogen-activated protein-serine kinase p38 alpha	NP_001306	Q16539
523	NK120-1	p38a MAPK	Pan-specific	Mitogen-activated protein-serine kinase p38 alpha	NP_001306	Q16539
524	NK120-10	p38a MAPK	Pan-specific	Mitogen-activated protein-serine kinase p38 alpha	NP_001306	Q16539
525	NK120-2	p38a MAPK	Pan-specific	Mitogen-activated protein-serine kinase p38 alpha	NP_001306	Q16539
526	NK120-3	p38a MAPK	Pan-specific	Mitogen-activated protein-serine kinase p38 alpha	NP_001306	Q16539
527	NK120-4	p38a MAPK	Pan-specific	Mitogen-activated protein-serine kinase p38 alpha	NP_001306	Q16539
528	NK120-5	p38a MAPK	Pan-specific	Mitogen-activated protein-serine kinase p38 alpha	NP_001306	Q16539
529	NK120-7	p38a MAPK	Pan-specific	Mitogen-activated protein-serine kinase p38 alpha	NP_001306	Q16539
530	NK059-1	p38g MAPK (Erk6)	Pan-specific	Mitogen-activated protein-serine kinase p38 gamma (MAPK12)	NP_002960	P53778
531	NK059-2	p38g MAPK (Erk6)	Pan-specific	Mitogen-activated protein-serine kinase p38 gamma (MAPK12)	NP_002960	P53778
532	NN082	p53	Pan-specific	Tumor suppressor protein p53 (antigenNY-CO-13)	NP_000537	P04637
533	PN057-1	p53	S392	Tumor suppressor protein p53 (antigenNY-CO-13)	NP_000537	P04637
534	PN057-2	p53	S392	Tumor suppressor protein p53 (antigenNY-CO-13)	NP_000537	P04637
535	PN057-3	p53	S392	Tumor suppressor protein p53 (antigenNY-CO-13)	NP_000537	P04637
536	PN158	p53	S33	Tumor suppressor protein p53 (antigenNY-CO-13)	NP_000537	P04637
537	PN159	p53	S37	Tumor suppressor protein p53 (antigenNY-CO-13)	NP_000537	P04637
538	PN160	p53	S6	Tumor suppressor protein p53 (antigenNY-CO-13)	NP_000537	P04637
539	NN123	p73	Pan-specific	Tumor suppressor protein p73	NP_005418	O15350
540	NP008	PAC1	Pan-specific	Dual specificity MAP kinase protein phosphatase	NP_004409	Q05923
541	NN084	PACSIN1	Pan-specific	Protein kinase C + casein kinase substrate in neurons protein 1	NP_065855	Q9BY11
542	NK122	PAK1	Pan-specific	p21-activated kinase 1 (alpha) (serine/threonine-protein kinase PAK 1)	NP_002567	Q13153
543	NK122-2	PAK1	Pan-specific	p21-activated kinase 1 (alpha) (serine/threonine-protein kinase PAK 1)	NP_002567	Q13153
544	NK122-4	PAK1	Pan-specific	p21-activated kinase 1 (alpha) (serine/threonine-protein kinase PAK 1)	NP_002567	Q13153
545	PK130	PAK1	T212	p21-activated kinase 1 (alpha) (serine/threonine-protein kinase PAK 1)	NP_002567	Q13153
546	NK224	PAK1/2/3	Pan-specific	p21-activated kinase 1/2/3 (serine/threonine-protein kinase PAK 1/2/3)	NP_002567	Q13153
547	PK061	PAK1/2/3	S144/S141/S154	p21-activated kinase 1/2/3 (serine/threonine-protein kinase PAK 1/2/3)	NP_002567	Q13153
548	NK200	PAK2	Pan-specific	p21-activated kinase 2 (gamma) (serine/threonine-protein kinase PAK 2)	NP_002568.2	Q13177

549	NK200-2	PAK2	Pan-specific	p21-activated kinase 2 (gamma) (serine/threonine-protein kinase PAK 2)	NP_002568.2	Q13177
550	NK123	PAK3	Pan-specific	p21-activated kinase 3 (beta) (serine/threonine-protein kinase PAK 3)	NP_002569	O75914
551	NN085-1	PARP1	Pan-specific	Poly [ADP-ribose] polymerase 1 (ADPRT)	NP_001609	P09874
552	NN085-2	PARP1	Pan-specific	Poly [ADP-ribose] polymerase 1 (ADPRT)	NP_001609	P09874
553	PN058	Pax2	S394	Paired box protein 2	NP_003978	Q02962
554	NN086	Paxillin 1	Pan-specific	Paxillin 1	NP_002850	P49023
555	PN059	Paxillin 1	Y31	Paxillin 1	NP_002850	P49023
556	PN060-1	Paxillin 1	Y118	Paxillin 1	NP_002850	P49023
557	PN060-3	Paxillin 1	Y118	Paxillin 1	NP_002850	P49023
558	NN087	PCNA	Pan-specific	Proliferating cell nuclear antigen	NP_002583	P12004
559	NK125	PCTK1 (PCTAIRE1)	Pan-specific	PCTAIRE-1 protein-serine kinase	NP_148978	Q00536
560	PK063	PDGFRa	Y754	Platelet-derived growth factor receptor kinase alpha	NP_006197	P16234
561	PK065	PDGFRb	Y716	Platelet-derived growth factor receptor kinase beta	NP_032835	P09619
562	NN141-1	PDI	Pan-specific	Protein disulfide-isomerase	NP_000909.2	P07237
563	NK126-1	PDK1	Pan-specific	3-phosphoinositide-dependent protein-serine kinase 1	NP_002604	O15530
564	NK126-2	PDK1	Pan-specific	3-phosphoinositide-dependent protein-serine kinase 1	NP_002604	O15530
565	PK066	PDK1	S241	3-Phosphoinositide-dependent protein-serine kinase 1	NP_002604	O15530
566	PN061	PED15 (PEA15)	S116	Phosphoprotein-enriched in diabetes/astrocytes 15	NP_003759	Q15121
567	NN088	PERP	Pan-specific	p53-induced protein PIGPC1	NP_071404	Q9H230
568	NN089	PI3K p85/p55	Pan-specific	Phosphatidylinositol 3-kinase regulatory subunit alpha	NP_852664	P27986
569	PN127	PI3K p85/p55	Y467/Y199	Phosphatidylinositol 3-kinase regulatory subunit alpha	NP_852664	P27986
570	NN114	PI3KR4	Pan-specific	Phosphoinositide-3-kinase, regulatory subunit 4	NP_055417	Q99570
571	NK192	PI4KCB	Pan-specific	phosphatidylinositol 4-kinase, catalytic, beta polypeptide	NP_002642	Q5VWC1
572	NK209	PIP5K2a	Pan-specific	Phosphatidylinositol 4-phosphatase 5-kinase type 2 alpha	NP_005019.2	P48426
573	NK213	PITSLRE	Pan-specific	PITSLRE serine/threonine-protein kinase CDC2L1	NP_277021.1	P21127
574	NK127-1	PKA Ca/b	Pan-specific	cAMP-dependent protein-serine kinase catalytic subunit alpha/beta	NP_002721	P17612
575	PK067	PKA Ca/b	T198	cAMP-dependent protein-serine kinase catalytic subunit alpha/beta	NP_002721	P17612
576	PK068	PKA Cb	S339	cAMP-dependent protein-serine kinase catalytic subunit beta	NP_002722	P22694
577	NN116	PKA R1a	Pan-specific	cAMP-dependent protein-serine kinase type I-alpha regulatory chain	NP_002725	P10644
578	NK128	PKA R2a	Pan-specific	cAMP-dependent protein-serine kinase regulatory type 2 subunit alpha	NP_004148	P13861
579	PK069	PKA R2a	S99	cAMP-dependent protein-serine kinase regulatory type 2 subunit alpha	NP_523671	P13861
580	NK129	PKBa (Akt1)	Pan-specific	Protein-serine kinase B alpha	NP_005154	P31749
581	NK129-2	PKBa (Akt1)	Pan-specific	Protein-serine kinase B alpha	NP_005154	P31749
582	PK071-2	PKBa (Akt1)	T308	Protein-serine kinase B alpha	NP_005154	P31749
583	PK072-1	PKBa (Akt1)	S473	Protein-serine kinase B alpha	NP_005154	P31749
584	PK072-3	PKBa (Akt1)	S473	Protein-serine kinase B alpha	NP_005154	P31749
585	PK072-5	PKBa (Akt1)	S473	Protein-serine kinase B alpha	NP_005154	P31749
586	PK148	PKBa (Akt1)	Y474	Protein-serine kinase B alpha	NP_005154	P31749
587	NK130-1	PKBb (Akt2)	Pan-specific	Protein-serine kinase B beta	NP_001617	P31751
588	NK130-2	PKBb (Akt2)	Pan-specific	Protein-serine kinase B beta	NP_001617	P31751
589	NK130-3	PKBb (Akt2)	Pan-specific	Protein-serine kinase B beta	NP_001617	P31751
590	NK130-5	PKBb (Akt2)	Pan-specific	Protein-serine kinase B beta	NP_001617	P31751
591	NK130-6	PKBb (Akt2)	Pan-specific	Protein-serine kinase B beta	NP_001617	P31751
592	NK130-7	PKBb (Akt2)	Pan-specific	Protein-serine kinase B beta	NP_001617	P31751
593	NK131-1	PKBg (Akt3)	Pan-specific	Protein-serine kinase B gamma	NP_005456	Q9Y243
594	NK131-2	PKBg (Akt3)	Pan-specific	Protein-serine kinase B gamma	NP_005456	Q9Y243
595	NK201	PKC	Pan-specific	Protein-serine kinase C alpha	NP_002728	P17252
596	NK218	PKCh	Pan-specific	Protein kinase C eta type	NP_006246.2	P24723
597	NK132	PKCa	Pan-specific	Protein-serine kinase C alpha	NP_002728	P17252
598	PK073	PKCa	S657	Protein-serine kinase C alpha	NP_002728	P17252
599	PK074	PKCa/b2	T638/T641	Protein-serine kinase C alpha/beta 2	NP_002728	P17252
600	NK133	PKCb1	Pan-specific	Protein-serine kinase C beta 1	NP_002729	P05771
601	NK133-2	PKCb1	Pan-specific	Protein-serine kinase C beta 1	NP_002729	P05771
602	PK075	PKCb1/2	T500	Protein-serine kinase C beta 1/2	NP_997700	P05771
603	PK075-2	PKCb1/2	T500	Protein-serine kinase C beta 1/2	NP_997700	P05771
604	NK134-2	PKCb2	Pan-specific	Protein-serine kinase C beta 2	AAA60095	P05771-2
605	PK076-2	PKCb2	T642	Protein-serine kinase C beta 2	NP_002729	P05771
606	NK135	PKCd	Pan-specific	Protein-serine kinase C delta	NP_006245	Q05655
607	PK077-1	PKCd	Y313	Protein-serine kinase C delta	NP_006245	Q05655
608	PK077-2	PKCd	Y313	Protein-serine kinase C delta	NP_006245	Q05655
609	PK078	PKCd	T507	Protein-serine kinase C delta	NP_006245	Q05655

610	PK079-1	PKCd	S645	Protein-serine kinase C delta	NP_006245	Q05655
611	PK080	PKCd	S664	Protein-serine kinase C delta	NP_006245	Q05655
612	NK136	PKCe	Pan-specific	Protein-serine kinase C epsilon	NP_005391	Q02156
613	NK136-2	PKCe	Pan-specific	Protein-serine kinase C epsilon	NP_005391	Q02156
614	PK081-1	PKCe	S729	Protein-serine kinase C epsilon	NP_005391	Q02156
615	NK137	PKCg	Pan-specific	Protein-serine kinase C gamma	NP_002730	P05129
616	PK082-1	PKCg	T514	Protein-serine kinase C gamma	NP_002730	P05129
617	PK082-2	PKCg	T514	Protein-serine kinase C gamma	NP_002730	P05129
618	PK083	PKCg	T655	Protein-serine kinase C gamma	NP_002730	P05129
619	PK084	PKCg	T674	Protein-serine kinase C gamma	NP_002730.1	P05129
620	PK085	PKCh	T655	Protein-serine kinase C eta	NP_006246	P24723
621	NK138-1	PKCl/i	Pan-specific	Protein-serine kinase C lambda/iota	NP_002731	P41743
622	PK087	PKCl/i	T564	Protein-serine kinase C lambda/iota	NP_002731	P41743
623	NK142	PKCm (PKD)	Pan-specific	Protein-serine kinase C mu (Protein kinase D)	NP_002733	Q15139
624	PK092	PKCm (PKD)	S738+S742	Protein-serine kinase C mu (Protein kinase D)	NP_002733	Q15139
625	PK093-1	PKCm (PKD)	S910	Protein-serine kinase C mu (Protein kinase D)	NP_002733	Q15139
626	PK093-2	PKCm (PKD)	S910	Protein-serine kinase C mu (Protein kinase D)	NP_002733	Q15139
627	NK140	PKCq	Pan-specific	Protein-serine kinase C theta	NP_006248	Q04759
628	PK088	PKCq	T538	Protein-serine kinase C theta	NP_006248	Q04759
629	PK089-1	PKCq	S676	Protein-serine kinase C theta	NP_006248	Q04759
630	PK090-1	PKCq	S695	Protein-serine kinase C theta	NP_006248	Q04759
631	NK141	PKCz	Pan-specific	Protein-serine kinase C zeta	NP_002735	Q05513
632	PK091	PKCz/l	T410/T412	Protein-serine kinase C zeta/lambda	NP_002735	Q05513
633	NK143	PKG1	Pan-specific	Protein-serine kinase G1 (cGMP-dependent protein kinase)	NP_006249	Q13976
634	NK202	PKG1a	Pan-specific	cGMP-dependent protein kinase 1, alpha isozyme	NP_006249	Q13976
635	NK203	PKG1b	Pan-specific	cGMP-dependent protein kinase 1, beta isozyme	NP_006249.1	P14619
636	NN115	PKM2	Pan-specific	Pyruvate kinase, isozymes M1/M2	NP_872270	P14618
637	NK144-1	PKR1	Pan-specific	Double-stranded RNA-dependent protein-serine kinase (EIF2AK2)	NP_002750	P19525
638	PK132	PKR1	T446	Double-stranded RNA-dependent protein-serine kinase (EIF2AK2)	NP_002750	P19525
639	NN156	PLC R(PLCg2)	Pan-specific	1-phosphatidylinositol-4,5-bisphosphate phosphodiesterase gamma-2	NP_002652.2	P16885
640	PN144	PLCg1	Y783	1-phosphatidylinositol-4,5-bisphosphate phosphodiesterase gamma-1	NP_877963.1	P19174
641	PN165	PLCg1	Y771	1-phosphatidylinositol-4,5-bisphosphate phosphodiesterase gamma-1	NP_877963.1	P19174
642	PN143	PLC R(PLCg2)	Y753	1-phosphatidylinositol-4,5-bisphosphate phosphodiesterase gamma-2	NP_002652.2	P16885
643	NK145	Plk1	Pan-specific	Polo-like protein-serine kinase 1	NP_005021	P53350
644	PK117	Plk1	T210	Polo-like protein-serine kinase 1	NP_005021	P53350
645	NK146	Plk2	Pan-specific	Polo-like protein kinase 2 (serum -inducible kinase (SNK))	NP_006613	Q9NYY3
646	NK146-2	Plk2	Pan-specific	Polo-like protein kinase 2 (serum -inducible kinase (SNK))	NP_006613	Q9NYY3
647	NK147	Plk3	Pan-specific	Polo-like protein kinase 3 (cytokine- inducible kinase (CNK))	NP_004064	Q9H4B4
648	NP009	PP1/Ca	Pan-specific	Protein-serine phosphatase 1 - catalytic subunit - alpha isoform	NP_002699	P62136
649	NP009-2	PP1/Ca	Pan-specific	Protein-serine phosphatase 1 - catalytic subunit - alpha isoform	NP_002699	P62136
650	PP001	PP1/Ca	T320	Protein-serine phosphatase 1 - catalytic subunit - alpha isoform	NP_002699	P62136
651	NP010	PP1/Cb	Pan-specific	Protein-serine phosphatase 1 - catalytic subunit - beta isoform	NP_002700	P62140
652	NP010-2	PP1/Cb	Pan-specific	Protein-serine phosphatase 1 - catalytic subunit - beta isoform	NP_002700	P62140
653	NP011	PP1/Cg	Pan-specific	Protein-serine phosphatase 1 - catalytic subunit - gamma isoform	NP_002701	P36873
654	NP033	PP2A B' (B56)	Pan-specific	Protein-serine phosphatase 2A - B regulatory subunit - B56 alpha isoform	NP_001186685.1	Q15172
655	NP012	PP2A/Aa/b	Pan-specific	Protein-serine phosphatase 2A - A regulatory subunit - alpha and beta isoforms	NP_002707	P30153
656	NP035	PP2A/Bb	Pan-specific	Protein-serine phosphatase 2A - B regulatory subunit - beta isoform	NP_001120853.1	Q00005
657	NP032	PP2A/Bg2	Pan-specific	Protein-serine phosphatase 2A - B regulatory subunit - gamma isoform	NP_001193923.1	Q9Y2T4
658	NP013-NP014	PP2A/Ca	Pan-specific	Protein-serine phosphatase 2A - catalytic subunit - alpha isoform	NP_002706	P67775
659	NP015	PP2B/Aa	Pan-specific	Protein-serine phosphatase 2B - catalytic subunit - alpha isoform	NP_000935	Q08209
660	NP016-NP031	PP2Ca	Pan-specific	Protein-serine phosphatase 2C - catalytic subunit - alpha	NP_066283	P35813
661	NP012-2	PP2A/Aa/b	Pan-specific	Protein-serine phosphatase 2A - A regulatory subunit - alpha and beta isoforms	NP_002707	P30153
662	NP018	PP2Cd	Pan-specific	Protein-serine phosphatase 2C - catalytic subunit - delta isoform	NP_110395	O15297
663	NP019	PP4/A'2	Pan-specific	Protein-serine phosphatase 4 - regulatory subunit (PPX/A'2)	NP_005125	Q8TF05
664	NP020	PP4C	Pan-specific	Protein-serine phosphatase X - catalytic subunit (PPX/C)	NP_002711	P60510
665	NP020-2	PP4C	Pan-specific	Protein-serine phosphatase X - catalytic subunit (PPX/C)	NP_002711	P60510
666	NP021	PP5C	Pan-specific	Protein-serine phosphatase 5 - catalytic subunit (PPT)	NP_006238	P53041
667	NP021-2	PP5C	Pan-specific	Protein-serine phosphatase 5 - catalytic subunit (PPT)	NP_006238	P53041
668	NP022	PP6C	Pan-specific	Protein-serine phosphatase 6 - catalytic subunit (PPVC)	NP_002712	O00743
669	PN062	PRAS40	T246	Proline-rich Akt substrate 40 kDa (Akt1S1)	NP_115751	Q96B36
670	NK148	PRK1 (PKN1)	Pan-specific	Protein kinase C-related protein-serine kinase 1	NP_002732	Q16512

671	PK095-PK096	PRK1 (PKN1)	T774	Protein kinase C-related protein-serine kinase 1	NP_002732	Q16512
672	NK149	PRK2 (PKN2)	Pan-specific	Protein kinase C-related protein-serine kinase 2	NP_006247	Q16513
673	NK149-2	PRK2 (PKN2)	Pan-specific	Protein kinase C-related protein-serine kinase 2	NP_006247	Q16513
674	NK150	PRKAB1	Pan-specific	5'-AMP-activated protein kinase (AMPK), beta-1 regulatory subunit	NP_006244	Q9Y478
675	NK151	WNK4 (PRKWNK4)	Pan-specific	Putative protein-serine kinase WNK4	NP_115763	Q96J92
676	PN104	Progesterone Receptor	S294	Progesterone receptor	NP_000917	P06401
677	NK152	PRP4K	Pan-specific	Protein-serine kinase PRP4 homolog	NP_003904	Q13523
678	NN142	PSD-95	Pan-specific	Disks large homolog 4	NP_001356.1	P78352
679	NK204	PSTAIRE	Pan-specific	PSTAIRE	NA	NA
680	NP023	PTEN	Pan-specific	Phosphatidylinositol-3,4,5-trisphosphate 3-phosphatase and protein phosphatase and tensin homolog deleted on chromosome 10	NP_000305	P60484
681	NP023-2	PTEN	Pan-specific	Phosphatidylinositol-3,4,5-trisphosphate 3-phosphatase and protein phosphatase and tensin homolog deleted on chromosome 10	NP_000305	P60484
682	PP003	PTEN	S380+T382+S385	Phosphatidylinositol-3,4,5-trisphosphate 3-phosphatase and protein phosphatase and tensin homolog deleted on chromosome 10	NP_000305	P60484
683	PP006	PTEN	S380+T382+T383	Phosphatidylinositol-3,4,5-trisphosphate 3-phosphatase and protein phosphatase and tensin homolog deleted on chromosome 10	NP_000305	P60484
684	PP006-1	PTEN	S380+T382+T383	Phosphatidylinositol-3,4,5-trisphosphate 3-phosphatase and protein phosphatase and tensin homolog deleted on chromosome 10	NP_000305	P60484
685	NP024	PTP1B	Pan-specific	Protein-tyrosine phosphatase 1B (PTPN1)	NP_002818	P18031
686	NP025	PTP1C	Pan-specific	Protein-tyrosine phosphatase 1C (SHP1, SHPTP1, PTPN6)	NP_002822	P29350
687	NP026	PTP1D	Pan-specific	Protein-tyrosine phosphatase 1D (SHP2, SHPTP2, Syp, PTP2C, PTPN11)	NP_002825	Q06124
688	NP026-2	PTP1D	Pan-specific	Protein-tyrosine phosphatase 1D (SHP2, SHPTP2, Syp, PTP2C, PTPN11)	NP_002825	Q06124
689	PP004	PTP1D	S580	Protein-tyrosine phosphatase 1D (SHP2, SHPTP2, Syp, PTP2C, PTPN11)	NP_002825	Q06124
690	NP036	PTPD1	Pan-specific	Protein-tyrosine phosphatase non-receptor type 21	NP_008970.2	Q16825
691	NP027	PTP-PEST	Pan-specific	Protein tyrosine phosphatase, non-receptor type 12	NP_001124480.1	Q05209
692	NK153	PyDK2 (PDHK2)	Pan-specific	Pyruvate dehydrogenase kinase isoform 2	NP_002602	Q15119
693	NK154	Pyk2	Pan-specific	Protein-tyrosine kinase 2	NP_004094	Q14289
694	PK097-3	Pyk2	Y579	Protein-tyrosine kinase 2	NP_004094	Q14289
695	NN150	Rab5	Pan-specific	Ras-related protein Rab-5A	NP_004153.2.	P20339
696	NN092-1	Rac1	Pan-specific	Ras-related C3 botulinum toxin substrate 1	NP_001782	P63000
697	PN063	Rac1	S71	Ras-related C3 botulinum toxin substrate 1	NP_008839	P63000
698	PN064	Rad17	S656	Rad17 homolog	NP_579921	O75943
699	NK155-2	Raf1	Pan-specific	Raf1 proto-oncogene-encoded protein-serine kinase	NP_002871	P04049
700	NK155-3	Raf1	Pan-specific	Raf1 proto-oncogene-encoded protein-serine kinase	NP_002871	P04049
701	NK155-4	Raf1	Pan-specific	Raf1 proto-oncogene-encoded protein-serine kinase	NP_002871	P04049
702	PK098	Raf1	S259	Raf1 proto-oncogene-encoded protein-serine kinase	NP_002871	P04049
703	NK205	RafA (Araf)	Pan-specific	A-Raf proto-oncogene serine/threonine-protein kinase	NP_001645.1	P10398
704	NK205-2	RafA (Araf)	Pan-specific	A-Raf proto-oncogene serine/threonine-protein kinase	NP_001645.1	P10398
705	NK156	RafB (Braf)	Pan-specific	RafB proto-oncogene-encoded protein-serine kinase	NP_004324	P15056
706	NK156-2	RafB (Braf)	Pan-specific	RafB proto-oncogene-encoded protein-serine kinase	NP_004324	P15056
707	NN093	Rb	Pan-specific	Retinoblastoma-associated protein 1	NP_000312	P06400
708	PN065	Rb	T356	Retinoblastoma-associated protein 1	NP_000312	P06400
709	PN066	Rb	S612	Retinoblastoma-associated protein 1	NP_000312	P06400
710	PN067	Rb	S780	Retinoblastoma-associated protein 1	NP_000312	P06400
711	PN068	Rb	S807	Retinoblastoma-associated protein 1	NP_000312	P06400
712	PN069	Rb	S807+S811	Retinoblastoma-associated protein 1	NP_000312	P06400
713	PN070	Rb	T821	Retinoblastoma-associated protein 1	NP_000312	P06400
714	PN071	Rb	T826	Retinoblastoma-associated protein 1	NP_000312	P06400
715	PN113	Rb	S608	Retinoblastoma-associated protein 1	NP_000312	P06400
716	PN131-1	Rb	S795	Retinoblastoma-associated protein 1	NP_000312	P06400
717	NN170	RelB	Pan-specific	Transcription factor RelB	NP_006500.2	Q01201
718	PN151	RelB	S573	Transcription factor RelB	NP_006500.2	Q01201
719	NK157	RIP2/RICK	Pan-specific	Receptor-interacting serine/threonine-protein kinase 2 (RIPK2)	NP_003812	O43353
720	NK158	RIPK1	Pan-specific	Receptor-interacting protein-serine kinase 1	NP_003795	Q13546
721	NK159-1	ROKa (ROCK2)	Pan-specific	RhoA protein-serine kinase alpha	NP_004841	O75116
722	NK159-2	ROKa (ROCK2)	Pan-specific	RhoA protein-serine kinase alpha	NP_004841	O75116
723	NK160	ROKb (ROCK1)	Pan-specific	RhoA protein-serine kinase beta	NP_005397	Q13464
724	NK161	RONa	Pan-specific	Macrophage-stimulating protein receptor alpha chain	NP_002438	Q04912
725	NK162	ROR2	Pan-specific	ROR2 neurotrophic receptor-tyrosine kinase	NP_004551	Q01974
726	NK163	ROS	Pan-specific	Orosomucoid 1 receptor-tyrosine kinase	NP_002935	P08922
727	NK164	RSK1	Pan-specific	Ribosomal S6 protein-serine kinase 1	NP_002944	Q15418
728	NK164-2	RSK1	Pan-specific	Ribosomal S6 protein-serine kinase 1	NP_002944	Q15418
729	PK157	RSK1	S363	Ribosomal S6 protein-serine kinase 1	NP_002944	Q15418
730	PK158	RSK1	T348	Ribosomal S6 protein-serine kinase 1	NP_002944	Q15418
731	PK099	RSK1/2	S221/S227	Ribosomal S6 protein-serine kinase 1/2	NP_002944	Q15418

732	PK100	RSK1/2	S363/S369	Ribosomal S6 protein-serine kinase 1/2	NP_002944	Q15418
733	PK100-2	RSK1/2	S363/S369	Ribosomal S6 protein-serine kinase 1/2	NP_002944	Q15418
734	PK101-1	RSK1/2	S380/S386	Ribosomal S6 protein-serine kinase 1/2	NP_002944	Q15418
735	PK101-2	RSK1/2	S380/S386	Ribosomal S6 protein-serine kinase 1/2	NP_002944	Q15418
736	PK102	RSK1/2/3	T573/T577/T570	Ribosomal S6 protein-serine kinase 1/2/3	NP_002944	Q15418
737	PK103	RSK1/3	T359+S363/T356+S360	Ribosomal S6 protein-serine kinase 1/3	NP_002944	Q15418
738	NK165	RSK2	Pan-specific	Ribosomal S6 protein-serine kinase 2	NP_004577	P51812
739	NK167	RYK	Pan-specific	RYK tyrosine-protein kinase	NP_001005861.1	P34925
740	PN073	S6	S235	40S ribosomal protein S6	NP_001001	P62753
741	PK156	S6K	S424	p70 ribosomal protein-serine S6 kinase	NP_003152	P23443
742	PK166	S6K	S411	p70 ribosomal protein-serine S6 kinase	NP_003152	P23443
743	NK223	S6Kb1	Pan-specific	Ribosomal protein-serine S6 kinase beta 1	NP_003152	P23443
744	NK223-1	S6Kb1	Pan-specific	Ribosomal protein-serine S6 kinase beta 1	NP_003152	P23443
745	NK223-2	S6Kb1	Pan-specific	Ribosomal protein-serine S6 kinase beta 1	NP_003152	P23443
746	PK145	S6Kb1	T252	Ribosomal protein serine S6 kinase beta 1	NP_003152	P23443
747	PK146	S6Kb1	T444+S447	Ribosomal protein serine S6 kinase beta 1	NP_003152	P23443
748	PK147	S6Kb1	T412	Ribosomal protein serine S6 kinase beta 1	NP_003152	P23443
749	NK222	S6Kb2	Pan-specific	Ribosomal protein-serine S6 kinase beta 2	NP_003943	Q9UBS0
750	NN133	SG2NA	Pan-specific	Striatin-3	NP_001077362.1	Q13033
751	PN074	Shc1	Y349+Y350	SH2 domain-containing transforming protein 1	NP_003020	P29353
752	PN161	Shc1	Y349	SH2 domain-containing transforming protein 1	NP_003020	P29353
753	NN095	Smac/DIABLO	Pan-specific	Second mitochondria-derived activator of caspase	NP_620308	Q9NR28
754	PN183	Smad1	S465	Mothers against decapentaplegic homolog 1	NP_005891	Q15797
755	PN075	Smad1/5/8	S463+S465/S463+S465/S465+S467	Mothers against decapentaplegic homologs 1/5/8	NP_005891	Q15797
756	PN184	Smad2	S467	Mothers against decapentaplegic homolog 2	NP_005891	Q15796
757	PN185	Smad2	T200	Mothers against decapentaplegic homolog 2	NP_005891	Q15796
758	NN096	Smad2/3	Pan-specific	SMA- and mothers against decapentaplegic homolog 2/3	NP_005892	Q15796
759	PN125	SMC1	S957	Structural maintenance of chromosomes protein 1A	NP_006297.2.	Q14683
760	PN177	SNCA	Y136	Alpha-synuclein	NP_000336.1	P37840
761	PN197	SNCA	S129	Alpha-synuclein	NP_000336.1	P37840
762	NN145	SOCS2	Pan-specific	Suppressor of cytokine signaling 2	NP_003868.1.	O14508
763	NN097	SOCS4	Pan-specific	Suppressor of cytokine signalling 4 (SOCS7)	NP_543143	Q8WXH5
764	NN098	SOD (Cu/Zn)	Pan-specific	Superoxide dismutase 1	NP_000445	P00441
765	NN068	SOD (Mn)	Pan-specific	Superoxide dismutase [Mn]	NP_000627	P04179
766	NN068-1	SOD (Mn)	Pan-specific	Superoxide dismutase [Mn]	NP_000627	P04179
767	NN099	SODD	Pan-specific	Silencer of death domains (Bcl2 associated athanogene 4 (BAG4))	NP_004865	O95429
768	PN077	SOX9	S181	SRY (sex determining region Y)-box 9 (campomelic dysplasia, autosomal sex-reversal)	NP_000337	P48436
769	NN100	SPHK1	Pan-specific	Sphingosine kinase 1	NP_892010	Q9NYA1
770	NN101	SPHK2	Pan-specific	Sphingosine kinase 2	NP_064511	Q9NRA0
771	NK172	Src	Pan-specific	Src proto-oncogene-encoded protein-tyrosine kinase	NP_005408	P12931
772	NK172-2	Src	Pan-specific	Src proto-oncogene-encoded protein-tyrosine kinase	NP_005408	P12931
773	NK172-3	Src	Pan-specific	Src proto-oncogene-encoded protein-tyrosine kinase	NP_005408	P12931
774	NK172-4	Src	Pan-specific	Src proto-oncogene-encoded protein-tyrosine kinase	NP_005408	P12931
775	PK107	Src	Y419	Src proto-oncogene-encoded protein-tyrosine kinase	NP_005408	P12931
776	PK108	Src	Y530	Src proto-oncogene-encoded protein-tyrosine kinase	NP_005408	P12931
777	NN102-NN124	STAT1a	Pan-specific	Signal transducer and activator of transcription 1 alpha	NP_009330	P42224
778	NN102-NN124-2	STAT1a	Pan-specific	Signal transducer and activator of transcription 1 alpha	NP_009330	P42224
779	NN139	STAT1a	Pan-specific	Signal transducer and activator of transcription 1 alpha	NP_009330	P42224
780	PN078-PN135	STAT1a	S727	Signal transducer and activator of transcription 1 alpha	NP_009330	P42224
781	PN079-PN136-1	STAT1a	Y701	Signal transducer and activator of transcription 1 alpha	NP_009330	P42224
782	NN103	STAT2	Pan-specific	Signal transducer and activator of transcription 2	NP_005410	P52630
783	PN080	STAT2	Y690	Signal transducer and activator of transcription 2	NP_005410	P52630
784	NN104	STAT3	Pan-specific	Signal transducer and activator of transcription 3 (acute phase response factor)	NP_003141	P40763
785	NN104-2	STAT3	Pan-specific	Signal transducer and activator of transcription 3 (acute phase response factor)	NP_003141	P40763
786	PN081-1	STAT3	S727	Signal transducer and activator of transcription 3	NP_003141	P40763
787	PN082	STAT3	Y705	Signal transducer and activator of transcription 3	NP_003141	P40763
788	PN082-1	STAT3	Y705	Signal transducer and activator of transcription 3	NP_003141	P40763
789	NN117	STAT4	Pan-specific	Signal transducer and activator of transcription 4 (acute phase response factor)	NP_003142	Q14765
790	NN105	STAT5A	Pan-specific	Signal transducer and activator of transcription 5A	NP_003143	P42229
791	PN083	STAT5A	Y694	Signal transducer and activator of transcription 5A	NP_003143	P42229
792	PN083-1	STAT5A	Y694	Signal transducer and activator of transcription 5A	NP_003143	P42229

793	PN119	STAT5A	S780	Signal transducer and activator of transcription 5A	NP_003143	P42229
794	NN106	STAT5B	Pan-specific	Signal transducer and activator of transcription 5B	NP_036580	P51692
795	NN107	STAT6	Pan-specific	Signal transducer and activator of transcription 6	NP_003144	P42226
796	NN108	STI1	Pan-specific	Stress induced phosphoprotein 1 (Hsc70/Hsp90 organizing protein (Hop))	NP_006810	P31948
797	NK173	STK33	Pan-specific	FLJ35932 protein-serine kinase	NP_112168	Q8NEF5
798	NN134	Striatin	Pan-specific	Striatin	NP_003153.2.	O43815
799	NK174	Syk	Pan-specific	Spleen protein-tyrosine kinase	NP_003168	P43405
800	PK159	Syk	Y323	Spleen protein-tyrosine kinase	NP_003168	P43405
801	NN171	Synapsin 1	Pan-specific	Synapsin 1 isoform Ia	NP_008881	P17600
802	PN084	Synapsin 1	S9	Synapsin 1 isoform Ia	NP_008881	P17600
803	NK175-2	TAK1	Pan-specific	TGF-beta-activated protein-serine kinase 1	NP_663306	O43318
804	NK175-3	TAK1	Pan-specific	TGF-beta-activated protein-serine kinase 1	NP_663306	O43318
805	NK175-4	TAK1	Pan-specific	TGF-beta-activated protein-serine kinase 1	NP_663306	O43318
806	NK175-5	TAK1	Pan-specific	TGF-beta-activated protein-serine kinase 1	NP_663306	O43318
807	PN085	Tau	S516	Microtubule-associated protein tau	NP_005901	P10636
808	PN086	Tau	S516+S519	Microtubule-associated protein tau	NP_005901	P10636
809	PN090	Tau	S713	Microtubule-associated protein tau	NP_005901	P10636
810	PN090-2	Tau	S713	Microtubule-associated protein tau	NP_005901	P10636
811	PN091	Tau	S717	Microtubule-associated protein tau	NP_005901	P10636
812	PN092	Tau	S721	Microtubule-associated protein tau	NP_005901	P10636
813	PN106	Tau	S519	Microtubule-associated protein tau	NP_005901	P10636
814	PN107	Tau	S739	Microtubule-associated protein tau	NP_005901	P10636
815	PN121	Tau	T522	Microtubule-associated protein tau	NP_005901	P10636
816	PN122	Tau	T548	Microtubule-associated protein tau	NP_005901	P10636
817	NK220-1	TBK1	Pan-specific	Serine/threonine-protein kinase TBK1	NP_037386	Q9UHD2
818	NK220-2	TBK1	Pan-specific	Serine/threonine-protein kinase TBK1	NP_037386	Q9UHD2
819	NK176	TEK (TIE2)	Pan-specific	Angiopoietin-1 receptor-tyrosine kinase	NP_444515	Q02763
820	NK177	Tlk1	Pan-specific	Tousled-like protein-serine kinase 1	NP_036422	Q9UKI8
821	NP034	TPIPb	Pan-specific	Phosphatidylinositol-3,4,5-trisphosphate 3-phosphatase TPTE2	NP_001135440.1	Q6XPS3
822	NN110	TRADD	Pan-specific	Tumor necrosis factor receptor type 1 associated DEATH domain protein	NP_003789	Q15628
823	NN111	Trail	Pan-specific	Tumor necrosis factor-related apoptosis-inducing ligand	NP_003801	P50591
824	NK178	TrkA	Pan-specific	Nerve growth factor (NGF) receptor-tyrosine kinase	NP_002520	P04629
825	NK179	TrkB	Pan-specific	BNDF/NT3/4/5 receptor- tyrosine kinase	NP_006171	Q16620
826	PK160	TrkB	Y706	BNDF/NT3/4/5 receptor- tyrosine kinase	NP_006171	Q16620
827	NK180	TTK	Pan-specific	Dual specificity protein kinase	AAA61239.1	P33981
828	NK181	Tyk2	Pan-specific	Protein-tyrosine kinase 2 (Jak-related)	NP_003322	P29597
829	NK181-2	Tyk2	Pan-specific	Protein-tyrosine kinase 2 (Jak-related)	NP_003322	P29597
830	NK183-1	Tyro10 (DDR2)	Pan-specific	Neurotrophic receptor-tyrosine kinase of discoidin domain receptor family, member 2 precursor	NP_006173	Q16832
831	NK183-2	Tyro10 (DDR2)	Pan-specific	Neurotrophic receptor-tyrosine kinase of discoidin domain receptor family, member 2 precursor	NP_006173	Q16832
832	PN093-1	Tyrosine Hydroxylase	S70	Tyrosine hydroxylase isoform a	NP_954986	P07101
833	PN109	Tyrosine Hydroxylase	S18	Tyrosine hydroxylase isoform a	NP_954986	P07101
834	NN176	VEGF-C	Pan-specific	Vascular endothelial growth factor C	NP_005420.1	P49767
835	PK161	VEGFR2 (KDR)	Y1059	Vascular endothelial growth factor receptor-tyrosine kinase 2 (KDR)	NP_002244	P35968
836	PK133	VEGFR2 (KDR)	Y1214	Vascular endothelial growth factor receptor-tyrosine kinase 2 (KDR)	NP_002244	P35968
837	NP030	VHR	Pan-specific	Dual specificity protein phosphatase 3	NP_004081	P51452
838	PN094	Vimentin	S34	Vimentin	NP_003371	P08670
839	NK184	Vrk1	Pan-specific	Vaccinia related protein-serine kinase 1	NP_003375	Q99986
840	NK185	Wee1	Pan-specific	Wee1 protein-tyrosine kinase	NP_003381	P30291
841	NP037	WIP1	Pan-specific	Protein phosphatase 1D	NP_003611.1.	O15297
842	NK186	Yes	Pan-specific	Yamaguchi sarcoma proto-oncogene-encoded tyrosine kinase	NP_005424	P07947
843	NK186-2	Yes	Pan-specific	Yamaguchi sarcoma proto-oncogene-encoded tyrosine kinase	NP_005424	P07947
844	NK214	YSK1	Pan-specific	Serine/threonine-protein kinase 25	NP_006365.2	O00506
845	NK187	ZAP70	Pan-specific	Zeta-chain (TCR) associated protein-tyrosine kinase, 70 kDa	NP_003168	P43403
846	NK187-2	ZAP70	Pan-specific	Zeta-chain (TCR) associated protein-tyrosine kinase, 70 kDa	NP_003168	P43403
847	PK109	ZAP70/Syk	Y319/Y352	Zeta-chain (TCR) associated protein-tyrosine kinase, 70 kDa/Spleen protein-tyrosine kinase	NP_001070	P43403
848	CN003	pThr(MmAb)	pThr	pThr(MmAb)	NA	NA
849	NK188-1	ZIPK	Pan-specific	ZIP kinase (death associated protein-serine kinase 3 (DAPK3))	NP_001339	O43293
850	NK188-2	ZIPK	Pan-specific	ZIP kinase (death associated protein-serine kinase 3 (DAPK3))	NP_001339	O43293
851	CN001	Actin	Pan-specific	Actin	NP_001092.1	P60709
852	CN002	Tubulin	Pan-specific	Tubulin	NP_006000.2	Q71U36
853	CN004	pThr(RpAb)	pThr	pThr(RpAb)	NA	NA

854	CN005	4G10		pTyr	4G10	NA	NA

Supplementary Table 2A - Antibody list of proteins with significant differential expression between shGRPR and scrambled in LNCaP.

No.	Antibody Codes	Target Protein Name	Phospho Site (Human)	Full Target Protein Name	Refseq	Uniprot Link	Average Z-ratio
89	NP001	CD45	Pan-specific	Leukocyte common antigen CD45 receptor-tyrosine phosphatase (LCA, T200)	NA	NA	-2,12
666	NP021	PP5C	Pan-specific	Protein-serine phosphatase 5 - catalytic subunit (PPT)	NP_005914	Q99683	-2,10
87	PN018	Caveolin 2	S36	Caveolin 2	NP_005148	P00519	-1,92
408	NK092-2	Lck	Pan-specific	Lymphocyte-specific protein-tyrosine kinase	NP_005772	Q07912	-1,83
73	NN013	CASP3	Pan-specific	Caspase 3 (apopain, cysteine protease CPP32)	NP_003397	P63104	-1,67
76	NN017	CASP7	Pan-specific	Caspase 7 (ICE-like apoptotic protease 3 (ICE-LAP3), Mch3)	NP_004086	Q13541	-1,58
646	NK146-2	Plk2	Pan-specific	Polo-like protein kinase 2 (serum -inducible kinase (SNK))	NP_004032	P49407	-1,57
844	NK214	YSK1	Pan-specific	Serine/threonine-protein kinase 25	NP_001871	P15336	-1,53
442	NK100-1	MEK2 (MAP2K2)	Pan-specific	MAPK/ERK protein-serine kinase 2 (MKK2)	NP_631897	Q96Q40	-1,48
419	NK097	MAPKAPK2	Pan-specific	Mitogen-activated protein kinase-activated protein kinase 2	NP_004199	O95831	-1,40
422	PN049-PN112-2	MAPKAPK2a	T334	Mitogen-activated protein kinase-activated protein kinase 2 alpha	NP_001616	P54819	-1,39
81	PN167	Catenin b	Y333	Catenin (cadherin-associated protein) beta 1	NP_004086	Q13541	-1,38
398	NN153	KDEL receptor 1	Pan-specific	ER lumen protein retaining receptor 1	NA	NA	-1,35
88	PN171	Cbl	Y700	Signal transduction protein CBL	NP_000655	Q13085	-1,35
658	NP013-NP014	PP2A/Ca	Pan-specific	Protein-serine phosphatase 2A - catalytic subunit - alpha isoform	NP_005914	Q99683	-1,35
639	NN156	PLC R(PLCg2)	Pan-specific	1-phosphatidylinositol-4,5-bisphosphate phosphodiesterase gamma-2	NP_004032	P49407	-1,33
828	NK181	Tyk2	Pan-specific	Protein-tyrosine kinase 2 (Jak-related)	NP_005914	Q99683	-1,32
83	NN021-1	Catenin b1	Pan-specific	Catenin (cadherin-associated protein) beta 1	NP_004086	Q13541	-1,32
464	NK106-2	MEK7 (MAP2K7)	Pan-specific	MAPK/ERK protein-serine kinase 7 (MKK7)	NP_006242	Q13131	-1,31
615	NK137	PKCg	Pan-specific	Protein-serine kinase C gamma	NP_000475.1	P05067	-1,27
428	NK099-1	MEK1 (MAP2K1)	Pan-specific	MAPK/ERK protein-serine kinase 1 (MKK1)	NP_004295.2	Q9UM73	-1,25
412	PK041	Lck	Y505	Lymphocyte-specific protein-tyrosine kinase	NP_058432	P35611	-1,22
84	NN167	Caveolin 1	Pan-specific	Caveolin 1	NP_004086	Q13541	-1,21
846	NK187-2	ZAP70	Pan-specific	Zeta-chain (TCR) associated protein-tyrosine kinase, 70 kDa	NP_001871	P15336	-1,21
584	PK072-3	PKBa (Akt1)	S473	Protein-serine kinase B alpha	NP_055093	O95757	-1,20
612	NK136	PKCe	Pan-specific	Protein-serine kinase C epsilon	NP_002145.3	P34932	-1,20

486	NK113-3	MST1	Pan-specific	Mammalian STE20-like protein-serine kinase 1 (KRS2)	NP_006273	Q13043	-1,20
541	NN084	PACSIN1	Pan-specific	Protein kinase C + casein kinase substrate in neurons protein 1	NP_065855	Q9BY11	1,22
8	NN135-1	Acetylated Lysine	Pan-specific	Acetylated Lysine	NA	NA	1,42
513	NN075	NT5E	Pan-specific	Ecto-5'-nucleotidase (CD73 antigen)	NP_002517	P21589	1,56
672	NK149	PRK2 (PKN2)	Pan-specific	Protein kinase C-related protein-serine kinase 2	NP_006247	Q16513	1,58
504	NN071	NFkappaB p65	Pan-specific	NF-kappa-B p65 nuclear transcription factor	NP_003989	Q04206	1,59
508	NK207	NIK (MAP3K14)	Pan-specific	NF-kappa beta-inducing kinase	NP_003945.2	Q99558	1,71
512	PN055-1	NR1	S896	N-methyl-D-aspartate (NMDA) glutamate receptor 1 subunit zeta	NP_000823	Q05586	1,73
527	NK120-4	p38a MAPK	Pan-specific	Mitogen-activated protein-serine kinase p38 alpha	NP_001306	Q16539	1,94
509	NK212	NLK	Pan-specific	Serine/threonine protein kinase NLK	NP_057315.3	Q9UBE8	2,02
530	NK059-1	p38g MAPK (Erk6)	Pan-specific	Mitogen-activated protein-serine kinase p38 gamma (MAPK12)	NP_002960	P53778	2,20
506	PN156	NFkappaB p65	S529	NF-kappa-B p65 nuclear transcription factor	NP_003989	Q04206	2,65
499	NK117-4	Nek2	Pan-specific	NIMA (never-in-mitosis)-related protein-serine kinase 2	NP_002488	P51955	2,79
495	PN187	NBS1	S343	Nijmegen breakage syndrome protein 1	NP_002476.2	O60934	2,96
498	NK117-3	Nek2	Pan-specific	NIMA (never-in-mitosis)-related protein-serine kinase 2	NP_002488	P51955	3,75

Supplementary Table 2B - Antibody list of proteins with significant differential expression between shGRPR and scrambled in VCaP.

No.	Antibody Codes	Target Protein Name	Phospho Site (Human)	Full Target Protein Name	Refseq	Uniprot Link	Average Z-ratio
288	PN038	Histone H3	S11	Histone H3.3	NP_003521	P84243	-2,29
111	NK026-5	CDK2	Pan-specific	Cyclin-dependent protein-serine kinase 2	NP_001789	P24941	-2,16
408	NK092-2	Lck	Pan-specific	Lymphocyte-specific protein-tyrosine kinase	NP_005347	P06239	-1,95
630	PK090-1	PKCq	S695	Protein-serine kinase C theta	NP_006248	Q04759	-1,90
646	NK146-2	Plk2	Pan-specific	Polo-like protein kinase 2 (serum -inducible kinase (SNK))	NP_006613	Q9NYY3	-1,90
615	NK137	PKCg	Pan-specific	Protein-serine kinase C gamma	NP_002730	P05129	-1,85
612	NK136	PKCe	Pan-specific	Protein-serine kinase C epsilon	NP_005391	Q02156	-1,81
613	NK136-2	PKCe	Pan-specific	Protein-serine kinase C epsilon	NP_005391	Q02156	-1,79
257	NK065	Fyn	Pan-specific	Fyn proto-oncogene-encoded protein-tyrosine kinase	NP_002028	P06241	-1,65
614	PK081-1	PKCe	S729	Protein-serine kinase C epsilon	NP_005391	Q02156	-1,64
606	NK135	PKCd	Pan-specific	Protein-serine kinase C delta	NP_006245	Q05655	-1,63
119	NK028-5	CDK5	Pan-specific	Cyclin-dependent protein-serine kinase 5	NP_004926	Q00535	-1,62
315	NN060-2	Hsp70	Pan-specific	Heat shock 70 kDa protein 1	NP_005336	P08107	-1,61
631	NK141	PKCz	Pan-specific	Protein-serine kinase C zeta	NP_002735	Q05513	-1,56

633	NK143	PKG1	Pan-specific	Protein-serine kinase G1 (cGMP-dependent protein kinase)	NP_006249	Q13976	-1,56
607	PK077-1	PKCd	Y313	Protein-serine kinase C delta	NP_006245	Q05655	-1,55
608	PK077-2	PKCd	Y313	Protein-serine kinase C delta	NP_006245	Q05655	-1,53
221	PK168-PK169	Erk1 (MAPK3)+ Erk2 (MAPK1)	Y204	Extracellular regulated protein-serine kinase 1 (p44 MAP kinase)+Extracellular regulated protein-serine kinase 2 (p42 MAP kinase)	AAA36142.1, NP_002736	P27361	-1,53
314	NN060	Hsp70	Pan-specific	Heat shock 70 kDa protein 1	NP_005336	P08107	-1,52
604	NK134-2	PKCb2	Pan-specific	Protein-serine kinase C beta 2	AAA60095	P05771-2	-1,51
228	NK206-3	Erk5 (MAPK7)	Pan-specific	Extracellular regulated protein-serine kinase 5 (Big MAP kinase 1 (BMK1))	NP_620602	Q13164	-1,48
470	NK108-2	MEKK2 (MAP3K2)	Pan-specific	MAPK/ERK kinase kinase 2	NP_006600.3	Q9Y2U5	-1,45
176	NN163	DDIT3(CHOP)	Pan-specific	DNA damage-inducible transcript 3 protein	NP_001181986.1	P35639	-1,44
478	PK056	MLK3	T277+S281	Mixed-lineage protein-serine kinase 3	NP_002410	Q16584	-1,43
828	NK181	Tyk2	Pan-specific	Protein-tyrosine kinase 2 (Jak-related)	NP_003322	P29597	-1,41
616	PK082-1	PKCg	T514	Protein-serine kinase C gamma	NP_002730	P05129	-1,38
584	PK072-3	PKBa (Akt1)	S473	Protein-serine kinase B alpha	NP_005154	P31749	-1,34
397	NP004	KAP	Pan-specific	Cyclin-dependent kinase associated phosphatase (CDK inhibitor 3, CIP2)	NP_005183	Q16667	-1,33
113	NK026-7	CDK2	Pan-specific	Cyclin-dependent protein-serine kinase 2	NP_001789	P24941	-1,33
393	PN048-2	Jun	S73	Jun proto-oncogene-encoded AP1 transcription factor	NP_002219	P05412	-1,30
396	PN163	Jun	T91	Jun proto-oncogene-encoded AP1 transcription factor	NP_002219	P05412	-1,29
638	PK132	PKR1	T446	Double-stranded RNA-dependent protein-serine kinase (EIF2AK2)	NP_002750	P19525	-1,29
625	PK093-1	PKCm (PKD)	S910	Protein-serine kinase C mu (Protein kinase D)	NP_002733	Q15139	-1,29
464	NK106-2	MEK7 (MAP2K7)	Pan-specific	MAPK/ERK protein-serine kinase 7 (MKK7)	NP_005034	O14733	-1,28
842	NK186	Yes	Pan-specific	Yamaguchi sarcoma proto-oncogene-encoded tyrosine kinase	NP_005424	P07947	-1,27
398	NN153	KDEL receptor 1	Pan-specific	ER lumen protein retaining receptor 1	NP_006792.1	P24390	-1,27
579	PK069	PKA R2a	S99	cAMP-dependent protein-serine kinase regulatory type 2 subunit alpha	NP_523671	P13861	-1,24
211	PK013-1	ErbB2 (HER2)	Y1248	ErbB2 (Neu) receptor-tyrosine kinase	NP_004439	P04626	-1,21
76	NN017	CASP7	Pan-specific	Caspase 7 (ICE-like apoptotic protease 3 (ICE-LAP3), Mch3)	NP_01218	P55210	-1,20
486	NK113-3	MST1	Pan-specific	Mammalian STE20-like protein-serine kinase 1 (KRS2)	NP_006273	Q13043	-1,20
696	NN092-1	Rac1	Pan-specific	Ras-related C3 botulinum toxin substrate 1	NP_001782	P63000	1,20
562	NN141-1	PDI	Pan-specific	Protein disulfide-isomerase	NP_000909.2	P07237	1,22
707	NN093	Rb	Pan-specific	Retinoblastoma-associated protein 1	NP_000312	P06400	1,23
144	NN026	Cofilin 1	Pan-specific	Cofilin 1	NP_005498	P23528	1,24
153	PN023	CREB1	S129+S133	cAMP response element binding protein 1	NP_004370	P16220	1,25
47	NK012	BMX (Etk)	Pan-specific	Bone marrow X protein-tyrosine kinase	NP_001712	P51813	1,26
41	NN007	Bcl-xL	Pan-specific	Bcl2-like protein 1	NP_612815	Q07817	1,27
700	NK155-3	Raf1	Pan-specific	Raf1 proto-oncogene-encoded protein-serine kinase	NP_002871	P04049	1,27

795	NN107	STAT6	Pan-specific	Signal transducer and activator of transcription 6	NP_003144	P42226	1,28
21	NN121	Arrestin b1	Pan-specific	Arrestin beta 1	NP_004032	P49407	1,28
811	PN091	Tau	S717	Microtubule-associated protein tau	NP_005901	P10636	1,31
780	PN078-PN135	STAT1a	S727	Signal transducer and activator of transcription 1 alpha	NP_009330	P42224	1,35
166	NN030-1	Cyclin D1	Pan-specific	Cyclin D1 (PRAD1)	NP_444284	P24385	1,36
766	NN068-1	SOD (Mn)	Pan-specific	Superoxide dismutase [Mn]	NP_000627	P04179	1,43
733	PK100-2	RSK1/2	S363/S369	Ribosomal S6 protein-serine kinase 1/2	NP_002944	Q15418	1,43
796	NN108	STI1	Pan-specific	Stress induced phosphoprotein 1 (Hsc70/Hsp90 organizing protein (Hop))	NP_006810	P31948	1,43
270	NN048	Grp78	Pan-specific	Glucose regulated protein 78	NP_005338	P11021	1,47
823	NN111	Trail	Pan-specific	Tumor necrosis factor-related apoptosis-inducing ligand	NP_003801	P50591	1,48
2	NN166	4E-BP1	Pan-specific	Eukaryotic translation initiation factor 4E binding protein 1 (PHAS1)	NP_004086	Q13541	1,51
682	PP003	PTEN	S380+T382+S385	Phosphatidylinositol-3,4,5-trisphosphate 3-phosphatase and protein phosphatase and tensin homolog deleted on chromosome 10	NP_000305	P60484	1,51
57	NN136-2	Calnexin	Pan-specific	Calnexin	NP_001019820.1.	P27824	1,52
156	NN149-1	Crystallin aB	Pan-specific	Crystallin alpha B (heat-shock 20 kDa like-protein)	NP_001876	P02511	1,56
58	NN137-1	Calreticulin	Pan-specific	Calreticulin	NP_004334.1.	P27797	1,58
783	PN080	STAT2	Y690	Signal transducer and activator of transcription 2	NP_005410	P52630	1,62
776	PK108	Src	Y530	Src proto-oncogene-encoded protein-tyrosine kinase	NP_005408	P12931	1,67
159	PN025	Crystallin aB	S19	Crystallin alpha B (heat-shock 20 kDa like-protein)	NP_001876	P02511	1,74
356	PN043	Integrin a4	S1027	Integrin alpha 4 (VLA4)	NP_000876	P13612	1,76
686	NP025	PTP1C	Pan-specific	Protein-tyrosine phosphatase 1C (SHP1, SHPTP1, PTPN6)	NP_002822	P29350	1,81
138	NK037-1	CK1e	Pan-specific	Casein protein-serine kinase 1 epsilon	NP_001885	P49674	1,83
685	NP024	PTP1B	Pan-specific	Protein-tyrosine phosphatase 1B (PTPN1)	NP_002818	P18031	1,84
732	PK100	RSK1/2	S363/S369	Ribosomal S6 protein-serine kinase 1/2	NP_002944	Q15418	1,86
666	NP021	PP5C	Pan-specific	Protein-serine phosphatase 5 - catalytic subunit (PPT)	NP_006238	P53041	1,87
269	NN047	Grp75	Pan-specific	Glucose regulated protein 75	NP_004125	P38646	1,89
390	NN162	Jun	Pan-specific	Jun proto-oncogene-encoded AP1 transcription factor	NP_002219	P05412	2,01
788	PN082-1	STAT3	Y705	Signal transducer and activator of transcription 3	NP_003141	P40763	2,02
163	NN028	Cyclin A	Pan-specific	Cyclin A1	NP_003905	P78396	2,02
721	NK159-1	ROCK2	Pan-specific	RhoA protein-serine kinase alpha	NP_004841	O75116	2,05
662	NP018	PP2Cd	Pan-specific	Protein-serine phosphatase 2C - catalytic subunit - delta isoform	NP_110395	O15297	2,08
798	NN134	Striatin	Pan-specific	Striatin	NP_003153.2.	O43815	2,33
777	NN102-NN124	STAT1a	Pan-specific	Signal transducer and activator of transcription 1 alpha	NP_009330	P42224	2,75
720	NK158	RIPK1	Pan-specific	Receptor-interacting protein-serine kinase 1	NP_003795	Q13546	3,07
782	NN103	STAT2	Pan-specific	Signal transducer and activator of transcription 2	NP_005410	P52630	3,29

Supplementary Table 2C - Antibody list of proteins down-regulated between shGRPR and scrambled in both LNCaP and VCaP.

No.	Antibody Codes	Target Protein Name	Phospho Site (Human)	Full Target Protein Name	Refseq	Uniprot Link	Average Z-ratio
408	NK092-2	Lck	Pan-specific	Lymphocyte-specific protein-tyrosine kinase	NP_005772	Q07912	-1,95
646	NK146-2	Plk2	Pan-specific	Polo-like protein kinase 2 (serum -inducible kinase (SNK))	NP_004032	P49407	-1,90
612	NK136	PKCe	Pan-specific	Protein-serine kinase C epsilon	NP_002145.3	P34932	-1,81
828	NK181	Tyk2	Pan-specific	Protein-tyrosine kinase 2 (Jak-related)	NP_005914	Q99683	-1,41
584	PK072-3	PKBa (Akt1)	S473	Protein-serine kinase B alpha	NP_055093	O95757	-1,34
464	NK106-2	MEK7 (MAP2K7)	Pan-specific	MAPK/ERK protein-serine kinase 7 (MKK7)	NP_006242	Q13131	-1,28
398	NN153	KDEL receptor 1	Pan-specific	ER lumen protein retaining receptor 1	NA	NA	-1,27
76	NN017	CASP7	Pan-specific	Caspase 7 (ICE-like apoptotic protease 3 (ICE-LAP3), Mch3)	NP_004086	Q13541	-1,20
486	NK113-2	MST1	Pan-specific	Mammalian STE20-like protein-serine kinase 1 (KRS2)	NP_006273	Q13043	-1,20
